# (−)-Tarchonanthuslactone Exerts a Blood Glucose-Increasing Effect in Experimental Type 2 Diabetes Mellitus

**DOI:** 10.3390/molecules20035038

**Published:** 2015-03-19

**Authors:** Gabriela F. P. de Souza, Luiz F. T. Novaes, Carolina M. Avila, Lucas F. R. Nascimento, Licio A. Velloso, Ronaldo A. Pilli

**Affiliations:** 1 Laboratory of Cell Signaling, University of Campinas, UNICAMP, C.P. 6154, CEP 13084-761 Campinas, São Paulo, Brazil; E-Mails: hbee_quimica@yahoo.com.br (G.F.P.S.); lribeiro_bio@yahoo.com.br (L.F.R.N.); lavelloso.unicamp@gmail.com (L.A.V.); 2 Institute of Chemistry, University of Campinas, UNICAMP, C.P. 6154, CEP 13084-971 Campinas, São Paulo, Brazil; E-Mails: luiztnovaes@gmail.com (L.F.T.N.); santanacma@gmail.com (C.M.A.)

**Keywords:** tarchonanthuslactone, caffeic acid, diabetes

## Abstract

A number of studies have proposed an anti-diabetic effect for tarchonanthuslactone based on its structural similarity with caffeic acid, a compound known for its blood glucose-reducing properties. However, the actual effect of tarchonanthuslactone on blood glucose level has never been tested. Here, we report that, in opposition to the common sense, tarchonanthuslactone has a glucose-increasing effect in a mouse model of obesity and type 2 diabetes mellitus. The effect is acute and non-cumulative and is present only in diabetic mice. In lean, glucose-tolerant mice, despite a slight increase in blood glucose levels, the effect was not significant.

## 1. Introduction

Type 2 diabetes mellitus (T2DM) is a metabolic disease defined by the presence of chronic hyperglycemia due to a simultaneous development of insulin resistance and a relative defect of the pancreatic islet to secrete insulin [1]. It is currently known that obesity-related metabolic inflammation plays an important role both in the installation of insulin resistance and damaging of the pancreatic islets [1]. Therefore, it is expected that therapeutic methods aimed at reducing inflammation may impact positively on the control of glucose homeostasis [2]. Currently, there is only one report of a clinical trial showing the metabolic benefits of reducing inflammation in diabetes using salsalate, which is derived from the bark of the willow tree [3]. However, additional studies, using different animal models [4] have evaluated other natural compounds for their potential as therapy for this disease [5,6,7].

**Figure 1:**
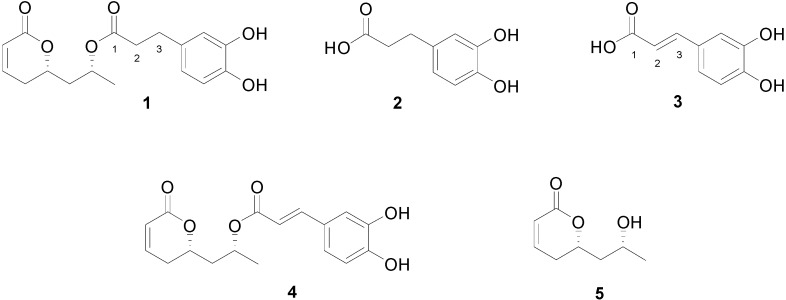
Chemical structures of compounds **1**–**5**.

Tarchonanthuslactone (**1**), an ester of dihydrocaffeic acid (**2**), was isolated in 1979 from the leaves of the tree *Tarchonanthus trilobus* [8]. The genus *Tarchonanthus* is found in southern Africa and is used in folk medicine. Traditional health practitioners have used *T. camphorate* for diabetes treatment; moreover, its anti-inflammatory and cytotoxic activities have been reported by van de Venter and coworkers [9]. Tarchonanthuslactone (**1**) has a privileged structure, presenting the usually bioactive α,β-unsaturated δ-lactone motif [10,11,12,13], which makes it a good target for new asymmetric synthetic approaches [14,15,16,17,18]. Interestingly, Hsu and coworkers [19] have reported that caffeic acid (**3**) has an antidiabetic effect and, because of the structural similarity between **1** and **3**, a number of reports have, thereafter, assigned a putative antidiabetic effect to **1** as well [14,15,16,17,18,20,21,22]. Here, we employed a mouse model of diet-induced diabetes to evaluate the effect of **1** and related compounds (Figure 1) on blood glucose levels. Such compounds may have effects on other metabolic parameters, however, we have focused on blood glucose levels.

## 2. Results and Discussion

Swiss mice belong to an outbread strain related to the diabetes prone Akr mouse [23]. Upon feeding on a high-fat diet (31% fat from lard) Swiss mice rapidly develop obesity accompained by insulin resistance and hyperglycemia [24]. In the present study, six-week old male Swiss mice were fed for eight weeks on a high-fat diet and then employed in the experiments.

**Figure 2:**
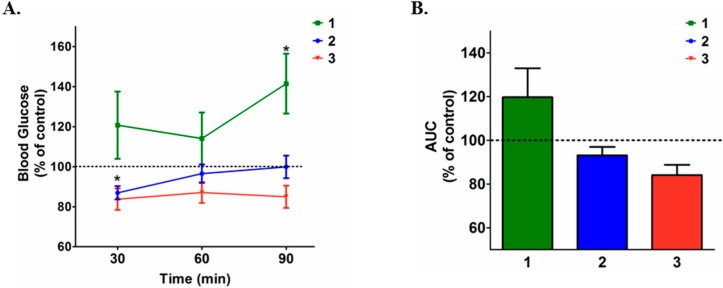
(**A**) Effect of a single intraperitoneal dose of **1**, **2** and **3** (3.0 mg/kg) on the blood glucose levels and (**B**) on area under the blood glucose response curve (AUC). Blood glucose levels and the AUC was calculated and expressed as percentage of saline treated group (mean ± SEM). * *p* < 0.05 *vs.* control.

Compound **3**, in the same dose as previously reported [19] was employed as a positive control. Six-hour fasting diabetic mice (median fasting blood glucose levels = 200 mg/dL) were randomly divided into three groups treated, by an intraperitoneal (ip) injection, with a single dose of either **1** (3.0 mg/kg), **2** (3.0 mg/kg) or **3** (3.0 mg/kg) and blood glucose levels were determined over the following 90 min. As depicted in Figure 2A,B, both **2** and **3** exerted a blood glucose-reducing effect, which was significant 30 min after the ip injection of the compounds but resulted in no significant reduction of the area under the glucose curve during the 90 min evaluation. Conversely, **1** exerted an unexpected blood glucose-increasing effect, which was significant at 90 min but resulted in no significant change in the area under the glucose curve during the 90 min evaluation. The effect of **3** obtained in our experiments matches the results reported previously [19], as maximal effect was obtained as early as 30 min after the injection of the compound. Since **2** presents similar glucose-reducing effect as **3**, we hypothesized that the presence of the double bond at C2-C3 (Figure 1), which is the only structural difference between **2** and **3**, would be involved in this biological effect. In order to test this hypothesis, we synthetized an analogue of **1** that possesses the C2-C3 double bond (**4**) and determined its effect on blood glucose levels.

**Figure 3:**
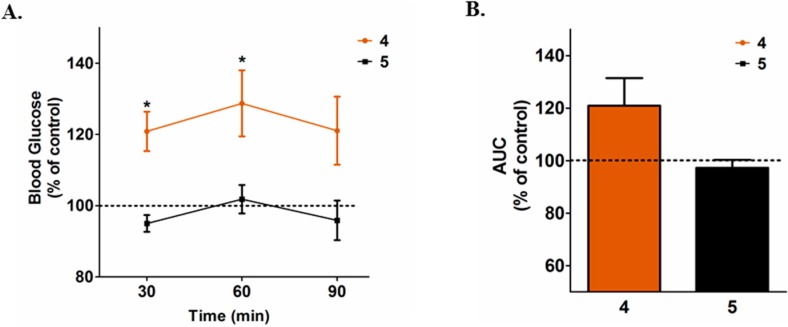
(**A**) Effect of a single intraperitoneal dose of **4** and **5** (3.0 mg/kg) on the blood glucose levels and (**B**) on area under the blood glucose response curve (AUC). Blood glucose levels and the AUC was calculated and expressed as percentage of saline treated group (mean ± SEM). * *p* < 0.05 *vs.* control.

As depicted in Figure 3A,B, in diabetic Swiss mice, **4** (3.0 mg/kg, ip) resulted in an even more potent glucose-increasing effect than **1**. Blood glucose levels were significantly higher than control at 30 and 60 min, leading to a significantly higher area under the glucose curve during the 90 min evaluation period. Thus, we propose that the presence/abscence of the C2-C3 double bond plays no role in either the glucose-reducing effect of **3** or the glucose-increasing effect of **1**.

Next, we hypothesized that the alcohol part of esther **1** could be responsible for the glucose-increasing effect produced by this compound. In order to test this hypothesis, diabetic Swiss mice were treated with the synthetic intermediate **5** (3.0 mg/kg, ip) and glucose levels were assessed. As depicted in Figure 3A,B, **5** had no effect on blood glucose levels in diabetic Swiss mice. Thus, we propose that neither the presence/absence of the C2-C3 double bond nor the alcohol part of **1** and **4** are required, *per se*, for the glucose regulatory effects of the compounds, rather, the whole molecules are required for the glucose-increasing effect.

**Figure 4:**
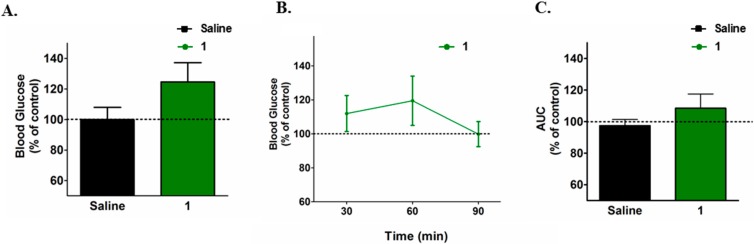
(**A**) Effect of **1** on fasting glycemia of chronically treated diabetic mice expressed as percentage of saline treated group; and (**B**) effect of a single intraperitoneal dose of **1** (3.0 mg/kg) on the blood glucose levels and (**C**) area under the blood glucose response curve (AUC) of lean treated mice expressed as percentage of lean saline treated group (mean ± SEM).

In addition, we evaluated the long-term and cumulative effect of **1** on blood glucose levels. For that, diabetic Swiss mice were treated three times a week, for four weeks with **1** (3.0 mg/kg per dose, ip). Two days after the last dose of the compound, the mice were submitted to a 6-hour fasting and blood glucose levels were determined. As depicted in Figure 4A, the prolonged treatment with **1** resulted in no significant change in fasting blood glucose level, suggesting that its blood-glucose increasing effect is acute and non-cumulative.

Finally, lean, non-diabetic Swiss mice, fed on chow (containing 4% fat), were accutely treated with **1** (3.0 mg/kg, ip) and blood glucose levels were determined over a 90 min time-frame. The median six-hour fasting glucose levels was 120 mg/dL and as depicted in Figure 4B, despite a slight increase in glucose levels at 60 min, **1** produced no statistically significant change in blood levels in lean mice.

In summary, **1** has no anti-diabetic effect as previously suggested [14,15,16,17,18]. In fact, despite its structural similarities with **2** and **3**, both of which capable of transiently reducing the blood glucose levels of diabetic animals, the accute treatment of diabetic mice with **1** results in a transient increase in blood glucose. Of note, the glucose-increasing effect of **1** is not due to particular features of the molecule, such as double bond between carbons 2 and 3 or the alcohol part of the molecule, but rather, to the whole molecule. The present study highlights how subtle structural modifications in chemical compounds can affect profoundly and unexpectedly its biological activity. The results described herein has potential impact on the design of more potent anti-diabetic compounds.

## 3. Experimental Section

### 3.1. Chemistry

#### 3.1.1. General Procedures

Starting materials and reagents were obtained from commercial sources and used as received unless otherwise specified. Dichloromethane was treated with calcium hydride and distilled before use. Tetrahydrofuran was treated with metallic sodium and benzophenone and distilled before use. Anhydrous reactions were carried out with continuous stirring under atmosphere of dry nitrogen. Progress of the reactions was monitored by thin-layer chromatography (TLC) analysis (silica gel 60 F^254^ on aluminum plates, Merck, Darmstadt, Hesse, Germany). ^1^H-NMR and ^13^C-NMR were recorded on Bruker 250, 400, 500 or 600 (Rheinstetten, Baden-Württemberg, Germany), the chemical shifts (δ) were reported in parts per million (ppm) relative to deuterated solvent as the internal standard (CDCl_3_ 7.26 ppm, 77.00 ppm, acetone-*d*_6_ 2.05 ppm, 29.92 ppm, methanol-*d*_4_ 3.31 ppm, 49.15 ppm), coupling constants (*J*) are in hertz (Hz). Peaks multiplicities are reported as follows: singlet (s), doublet (d), triplet (t), quartet (q), sextet (sext), multiplet (m), broad singlet (br s). Mass spectra were recorded on a Waters Xevo Q-Tof apparatus operating in electrospray mode (ES). Infrared spectra with Fourier transform (FT-IR) were recorded on a Therm Scientific Nicolet iS5, the principal absorptions are listed in cm^−1^. The values of optical rotation were measured at 25 °C in a polarimeter Perkin-Elmer 341, with sodium lamp, the measure is described as follow {[α]*_D_* (*c* = g/100 mL), solvent}.

#### 3.1.2. Experimental Procedures

*(R)-Butane-1,3-diol* (**ii**). To a suspension of LiAlH_4_ (200 mg, 5.27 mmol) in anhydrous THF (9.2 mL) at 0 °C, was added PHB (**i**, 602 mg), then the temperature increased until rt. The reaction was stirred for 90 min at this temperature, and for 5 h under reflux, and at rt again overnight. The reaction was cooled at 0 °C and was added with intervals 0.2 mL of water, 0.2 mL of aqueous solution of NaOH 10% and 0.6 mL of water. The reaction was filtered and the solid was washed with THF (3 × 25 mL), the organic phase was concentrated and the product was purified by column chromatography (SiO_2_, CHCl_3_/MeOH 95:5) to afford the corresponding diol **ii** (595 mg, 94%). Colorless oil. R*_f_* 0.67 (SiO_2_, CHCl_3_/MeOH 90:10). ^1^H-NMR δ (CDCl_3_, 250 MHz): 1.15 (d, *J* = 6.2 Hz, 3H), 1.61 (q, *J* = 5.5 Hz), 3.62–3.82 (m, 2H), 3.90–4.02 (m, 3H). ^13^C-NMR δ (CDCl_3_, 62.9 MHz): 23.6, 40.2, 60.8, 67.3. [α]*_D_* (*c* = 1.0, CHCl_3_): −21. Lit. [25]: [α]*_D_* (*c* = 1.66, CHCl_3_): −30.1.

*(R)-2,2,3,3,5,9,9,10,10-Nonamethyl-4,8-dioxa-3,9-disilaundecane* (**iii**). To a solution of the diol **ii** (1.00 g, 11.1 mmol, 1 eq.) in anhydrous CH_2_Cl_2_ (44 mL), was added imidazole (2.27 g, 33.3 mmol, 3 eq.) and TBSCl (4.18 g, 27.7 mmol, 2.5 eq.), the reaction was stirred for 6 h, and were added to it, 75 mL of water and 120 mL of EtOAc, the aqueous phase was extracted once with 50 mL of EtOAc, the organic phases were grouped, dried (Na_2_SO_4_), filtered and concentrated, the crude was purified by column chromatography (SiO_2_, hexanes/EtOAc 98:2) to afford **iii** (3.45 g, 98%). Colorless oil. R*_f_* 0.51 (SiO_2_, hexanes/EtOAc 98:2). ^1^H-NMR δ (CDCl_3_, 250 MHz): 0.04 (s, 6H), 0.05 (s, 6H), 0.89 (s, 18H), 1.14 (d, *J* = 6.2 Hz, 3H), 1.50–1.75 (m, 2H), 3.67 (t, *J* = 6.5 Hz, 2H), 3.97 (sext, *J* = 6.1 Hz, 1H). ^13^C-NMR δ (CDCl_3_, 62.9 MHz): −5.1 (2C), −4.6, −4.2, 18.3, 18.4, 24.2, 26.1 (3C), 26.1 (3C), 43.0, 60.2, 65.7. [α]*_D_* (*c* = 1.0, CHCl_3_): −20. IR (NaCl, cm^−1^): 1255, 1384, 1472, 2858, 2886, 2930, 2956. HRMS: [C_16_H_38_O_2_Si_2_+H]^+^ calculated 319.2489, observed 319.2471.

*(R)-3-((tert-Butyldimethylsilyl)oxy)butan-1-ol* (**iv**). To a solution of **iii** (2.54 g, 7.97 mmol, 1 eq.) in anhydrous THF (30 mL) at 0 °C, was added a solution composed by pyridine (20 mL) and HF•pyridine (8.8 mL) in THF (50 mL). The reaction was stirred at rt for 2.5 h. The reaction was neutralized with solution of NaHCO_3_ (150 mL) and it was stirred for other 10 min. The reaction contents were diluted with 150 mL of EtOAc and the aqueous phase was extracted with EtOAc (2 × 50 mL). The organic phases were combined, dried (MgSO_4_) and concentrated, the product was purified by column chromatography (SiO_2_, hexanes/EtOAc 75:25) to afford **iv** (895 mg, 55%). Colorless oil. R*_f_* 0.40 (SiO_2_, hexanes/EtOAc 80:20). ^1^H-NMR δ (CDCl_3_, 250 MHz): 0.06 (s, 3H), 0.06 (s, 3H), 0.87 (s, 9H), 1.17 (d, *J* = 6.2 Hz, 3H), 1.54–1.82 (m, 2H), 2.72 (br s, 1H), 3.63–3.85 (m, 2H), 4.00–4.14 (m, 1H). ^13^C-NMR δ (CDCl_3_, 62.9 MHz): −4.9, −4.3, 18.1, 23.6, 25.9 (3C), 40.7, 60.5, 68.3. [α]*_D_* (*c* = 1.0, CHCl_3_): −25. Lit. [26]: [α]*_D_* (*c* = 0.41, CHCl_3_): −17.8.

*(R)-3-((tert-Butyldimethylsilyl)oxy)butanal* (**v**). In a dried 100 mL round bottom flask, was added a solution of freshly distilled oxalyl chloride (0.74 mL, 8.59 mmol, 1.5 eq) dissolved in anhydrous CH_2_Cl_2_ (15 mL). The solution was cooled at −78 °C. After this, was added dropwise anhydrous DMSO (1.3 mL, 18.3 mmol, 3.2 eq.). The reaction stayed under stirring for 15 min at −78 °C, then the alcohol **iv** (1.17 g, 5.73 mmol, 1.0 eq.) dissolved in anhydrous CH_2_Cl_2_ (10 mL) was added to the mixture *via* cannula. The reaction was stirred for other period of 15 min, and freshly distilled triethylamine (3.9 mL, 28 mmol, 5.0 eq.) was added. After 30 min of stirring, the reaction contents were diluted in 20 mL of Et_2_O and 15 mL of aqueous solution of NH_4_Cl. The aqueous phase was extracted with Et_2_O (3 × 15 mL) and the organic phases were grouped, washed with brine, dried (MgSO_4_), filtered and concentrated. The product was purified by column chromatography (SiO_2_, hexanes/EtOAc 95:5) to afford **v** (1.00 g, 86%). Colorless oil. R*_f_* 0.57 (SiO_2_, hexanes/EtOAc 90:10). ^1^H-NMR δ (CDCl_3_, 250 MHz): 0.02 (s, 3H), 0.03 (s, 3H), 0.82 (s, 9H), 1.19 (d, *J* = 6.2 Hz, 3H), 2.35–2.60 (m, 2H), 4.31 (sext, *J* = 6.1 Hz, 1H), 9.74 (t, *J* = 2.3 Hz, 1H). ^13^C-NMR δ (CDCl_3_, 62.9 MHz): −4.9, −4.3, 18.0, 24.2, 25.8 (3C), 53.1, 64.6, 202.0. [α]*_D_* (*c* = 1.0, CHCl_3_): −15. Lit. [27]: *ent*-compound [α]*_D_* (*c* = 1.0, CHCl_3_): +14.

*(4S,6R)-6-((tert-Butyldimethylsilyl)oxy)hept-1-en-4-ol* (**vi**). In a 50 mL round bottom flask with a magnetic stir bar were added powdered activated molecular sieves 4 Å (2.5 g). After the addition of the molecular sieves the flask was heated under flow of N_2_. After this anhydrous CH_2_Cl_2_ (10 mL), *S*-BINOL (286 mg, 1.00 mmol, 0.2 eq.), TFA (1 μL) and Ti(O-*i*Pr)_4_ (150 μL) were added to the flask. The mixture was refluxed for 2 h, when it was observed a change of color from dark red to brown. After this period, the bath of oil was removed and temperature decrease until rt and the aldehyde **v** (986 mg, 4.87 mmol, 1.0 eq.) dissolved in anhydrous CH_2_Cl_2_ (10 mL) was added. The mixture was maintained under stirring for 15 min. The reaction was placed in a bath at −78 °C and allyltributylstannane (2.34 mL, 7.4 mmol, 1.5 eq.) was added slowly to the mixture. The reaction was maintained under stirring at −30 °C for 4 days. After this period, brine (40 mL) was added to the reaction and the temperature raised until rt. After 1 h the mixture was filtered. The aqueous phase was extracted with CH_2_Cl_2_ (3 × 50 mL). The organic phases were grouped, dried (Na_2_SO_4_), filtered and concentrated. The product was purified by column chromatography (SiO_2_, hexanes/EtOAc 95:5) to afford **vi** (524 mg, 44%) and aldehyde **v** (275 mg, 28%) was recovered. Colorless oil. R*_f_* 0.34 (SiO_2_, hexanes/EtOAc 90:10). *d.r.* 12:1 (*syn/anti*). ^1^H-NMR δ (CDCl_3_, 250 MHz): 0.07 (s, 3H), 0.08 (s, 3H), 0.86 (s, 9H), 1.15 (d, *J* = 6.2 Hz, 3H), 1.48–1.57 (m, 2H), 2.11–2.22 (m, 2H), 3.26 (br s, 1H), 3.73–3.85 (m, 1H), 3.97–4.10 (m, 1H), 5.00–5.11 (m, 2H), 5.80 (ddt, J = 16.9, 10.2, 7.1 Hz, 1H). ^13^C-NMR δ (CDCl_3_, 62.9 MHz): −4.7, −3.8, 18.0, 24.6, 25.9 (3C), 42.1, 45.3, 69.8, 70.6, 117.3, 135.0. [α]*_D_* (*c* = 1.0, CHCl_3_): −30. Lit. [28]: *ent*-compound [α]*_D_*_,literature_ (*c* = 0.76, CHCl_3_): +32.8.

*(4S,6R)-6-((tert-Butyldimethylsilyl)oxy)hept-1-en-4-yl acrylate* (**vii**). To a solution of alcohol **vi** (2.00 g, 1.0 eq.) in anhydrous CH_2_Cl_2_ (41 mL) at 0 °C, was added freshly distilled triethylamine (2.3 mL, 2.0 eq.), followed by slowly addition of acryloyl chloride (1.0 mL, 1.5 eq.). The reaction was stirred for 4 h at rt. After this period, brine (20 mL) and aqueous solution of Rochelle salt (20 mL) were added. The aqueous phase was extracted with EtOAc (3 × 50 mL). The organic phases were grouped, dried (Na_2_SO_4_), filtered and concentrated. The product was purified by column chromatography (SiO_2_, hexanes/EtOAc 95:5) to afford **vii** (1.80 g, 75%). Colorless oil. R*_f_* 0.30 (SiO_2_, hexanes/EtOAc 99:1). ^1^H-NMR δ (CDCl_3_, 600 MHz): 0.04 (s, 3H), 0.05 (s, 3H), 0.88 (s, 9H), 1.15 (d, *J* = 6.0 Hz, 3H), 1.63–1.68 (m, 1H), 1.82–1.88 (m, 1H), 2.30–2.43 (m, 2H), 3.85 (sext, *J* = 6.2 Hz, 1H), 5.04–5.11 (m, 3H), 5.71–5.79 (m, 1H), 5.80 (dd, *J* = 10.4, 1.3 Hz, 1H), 6.09 (dd, *J* = 17.3, 10.5 Hz, 1H), 6.38 (dd, *J* = 17.3, 1.3 Hz, 1H). ^13^C-NMR δ (CDCl_3_, 151 MHz): −4.7, −4.3, 18.2, 23.6, 26.0 (3C), 39.0, 43.7, 65.7, 71.1, 118.0, 128.9, 130.5, 133.5, 165.7. [α]*_D_* (*c* = 1.0, CHCl_3_): +13. IR (NaCl, cm^−1^): 1194, 1406, 1472, 1727, 2858, 2896, 2930, 2958. HRMS: [C_16_H_30_O_3_Si+H]^+^ calculated 299.2043, observed 299.2107.

*(S)-6-((R)-2-((tert-butyldimethylsilyl)oxy)propyl)-5,6-dihydro-2H-pyran-2-one* (**viii**). To a solution of the acrylate **vii** (1969 mg, 6.6 mmol, 1.0 eq.) in CH_2_Cl_2_ (540 mL) at temperature of 40–45 °C, Grubbs catalyst 1st generation (543 mg, 0.66 mmol, 0.1 eq.) was added. The mixture was refluxed for 4 h. After this time the solvent was evaporated. The product was purified by column chromatography (SiO_2_, hexanes/EtOAc 95:5) to afford **viii** (1.40 g, 80%). Brown oil. R*_f_* 0.34 (SiO_2_, hexanes/EtOAc 80:20). ^1^H-NMR δ (CDCl_3_, 500 MHz): 0.00 (s, 3H), 0.02 (s, 3H), 0.82 (s, 9H), 1.15 (d, *J* = 6.1 Hz, 3H), 1.64–1.71 (m, 1H), 1.96–2.03 (m, 1H), 2.26–2.41 (m, 2H), 4.03 (sext, *J* = 6.1 Hz, 1H), 4.49–4.56 (m, 1H), 5.93–5.98 (m, 1H), 6.84 (ddd, *J* = 9.8, 5.8, 2.7 Hz, 1H). ^13^C-NMR δ (CDCl_3_, 126 MHz): −4.8, −4.3, 18.0, 23.4, 25.8 (3C), 29.7, 44.2, 64.9, 75.5, 121.4, 145.1, 164.4. [α]*_D_* (*c* = 1.0, CHCl_3_): −91. Lit. [29]: [α]*_D_* (*c* = 0.84, CHCl_3_): −92.6.

*(S)-6-((R)-2-Hydroxypropyl)-5,6-dihydro-2H-pyran-2-one* (**5**). To a solution of **viii** (210 mg, 0.8 mmol, 1 eq.) in anhydrous THF (10 mL) at 0 °C, was added a solution composed by pyridine (1.3 mL) and HF•pyridine (0.6 mL) in THF (13 mL). The reaction was stirred at rt for 3 days. The reaction was neutralized with solution of NaHCO_3_ (30 mL). The reaction contents were diluted with 30 mL of ethyl acetate and the aqueous phase was extracted with EtOAc (3 × 30 mL). The product was purified by column chromatography (SiO_2_, EtOAc) to afford **5** (121 mg, quantitative yield). Colorless oil. R*_f_* 0.40 (SiO_2_, EtOAc). ^1^H-NMR δ (CDCl_3_, 500 MHz): 1.20 (d, *J* = 6.3 Hz, 3H), 1.67–1.74 (m, 1H), 1.92–2.00 (m, 1H), 2.30–2.44 (m, 2H), 2.72 (br s, 1H), 3.96–4.05 (m, 1H), 4.55–4.63 (m, 1H), 5.95 (dd, *J* = 9.9, 1.7 Hz, 1H), 6.87 (ddd, *J* = 9.3, 5.8, 2.6 Hz, 1H). ^13^C-NMR δ (CDCl_3_, 126 MHz): 23.7, 29.4, 43.5, 64.9, 76.8, 121.0, 145.7, 164.4. [α]*_D_* (*c* = 1.0, CHCl_3_): −128. Lit. [18]: [α]*_D_* (*c* = 0.17, CHCl_3_): −115.5.

*(E)-3-(3,4-bis((tert-Butyldimethylsilyl)oxy)phenyl)acrylic acid* (**ix**). Freshly distilled diisopropylethylamine (2.91 mL, 16.7 mmol, 5.0 eq) and *tert*-butyldimethylsilyl chloride (2.09 g, 13.9 mmol, 5.0 eq) were added to a suspension of caffeic acid (500 mg, 2.78 mmol, 1 eq) in anhydrous CH_2_Cl_2_ (3.4 mL) at 25 °C, and the mixture became a solution, it was stirred at 25 °C for 14 h. The mixture was diluted with EtOAc (15 mL), extracted with water (5 mL), aqueous solution of HCl 1 M (2 × 10 mL) and brine (10 mL), then the organic phase was dried (MgSO_4_) and concentrated to obtain an yellow oil. This oil was dissolved in THF (4 mL) and were added solid K_2_CO_3_ (400 mg) and water (0.7 mL), the reaction was stirred for 2 h. Upon completion the reaction contents were diluted with ethyl acetate (15 mL), extracted with water (10 mL), aqueous solution of HCl 1 M (10 mL) and brine (10 mL), then the organic phase was dried (MgSO_4_) and concentrated. The solid was submitted to *vacuo* (10 mbar) at 60 °C for 4 h, to obtain **ix** (1.078 g, 95%). Pale yellow solid, mp 157–160 °C. R*_f_* 0.40 (SiO_2_, hexanes/EtOAc 75:25), ^1^H-NMR δ (CDCl_3_, 250 MHz): 0.22 (s, 6H), 0.23 (s, 6H), 0.99 (s, 9H), 1.00 (s, 9H), 6.24 (d, *J* = 15.8 Hz, 1H), 6.81–6.87 (m, 1H), 7.01–7.08 (m, 2H), 7.67 (d, *J* = 16.0 Hz, 1H), ^13^C-NMR δ (CDCl_3_, 62.9 MHz): −4.0 (2C), −3.9 (2C), 18.6, 18.6, 26.0 (6C), 115.0, 120.8, 121.3, 122.9, 127.8, 147.2, 147.4, 150.1, 173.2.

*3-(3,4-Bis((tert-butyldimethylsilyl)oxy)phenyl)propanoic acid* (**x**). 40 mg of Pd/C (5% m/m) were added to a solution of acid **ix** (0.92 mmol) in ethyl acetate (5 mL). The heterogeneous mixture was stirred under hydrogen atmosphere for 3 h. The mixture was filtered through celite, and the solvent was removed under *vacuo* to afford the acid **x** (373 mg, 99%). pale yellow solid. mp 88–89 °C. R*_f_* 0.54 (SiO_2_, hexanes/EtOAc 75:25). ^1^H-NMR δ (CDCl_3_, 250 MHz): 0.18 (s, 12H), 0.98 (s, 18H), 2.62 (t, *J* = 7.5 Hz, 2H), 2.84 (t, *J* = 7.8 Hz, 2H), 6.60–6.78 (m, 3H). ^13^C-NMR δ (CDCl_3_, 62.9 MHz): −3.95 (4C), 18.6 (2C), 26.1 (6C), 30.1, 36.1, 121.2 (2C), 121.3, 133.4, 145.4, 146.8, 179.7.

*(*−*)-Tarchonanthuslactone* (**1**). To a solution of EDC•HCl (143 mg, 0.75 mmol, 2 eq.) and DMAP (50 mg, 0.38 mmol, 1 eq.) in anhydrous CH_2_Cl_2_ (5 mL) were added a solution of acid **x** (308 mg, 0.75 mmol, 2 eq.) and alcohol 5 (60 mg, 0.31 mmol, 1 eq.) in anhydrous CH_2_Cl_2_ (5 mL) at 25 °C, and the mixture was stirred at 25 °C for 1 day. Upon completion, the reaction contents were diluted in EtOAc (90 mL), and extracted with solution of HCl 0.5 M (40 mL). The organic phase was washed with aqueous solution of saturated NaHCO_3_ (30 mL), dried (MgSO_4_), and concentrated. The crude was dissolved in anhydrous THF (10 mL), and benzoic acid (231 mg, 1.9 mmol, 5 eq.) and solution of TBAF 1 M in THF (1.9 mL, 1.9 mmol, 5 eq.) were added at 0 °C. The reaction was stirred at this temperature for 30 min, then aqueous solution of saturated NaHCO_3_ (30 mL) was added, and the aqueous phase was extracted with EtOAc (2 × 60 mL). The organic phases were grouped, washed with brine (30 mL) and dried (MgSO_4_). The product was purified by column chromatography (SiO_2_, hexanes/EtOAc 60:40 to 30:70) to afford **1** (71 mg, 58%). Viscous colorless oil. R*_f_* 0.50 (SiO_2_, hexanes/EtOAc 30:70). ^1^H-NMR δ (CDCl_3_, 500 MHz): 1.22 (d, *J* = 6.3 Hz, 3H), 1.73 (ddd, *J* = 14.5, 6.9, 4.3 Hz, 1H), 2.06 (ddd, *J* = 14.6, 8.4, 6.4 Hz, 1H), 2.11–2.31 (m, 2H), 2.58 (t, *J* = 7.5 Hz, 2H), 2.80 (t, *J* = 7.2 Hz, 2H), 4.16–4.24 (m, 1H), 5.01–5.08 (m, 1H), 5.98 (dd, 9.8, 1.8 Hz, 1H), 6.55 (dd, *J* = 8.1, 1.8 Hz, 1H), 6.71 (d, *J* = 1.8 Hz, 1H), 6.73 (d, *J* = 7.9 Hz, 1H), 6.78–6.85 (m, 1H). ^13^C-NMR δ (CDCl_3_, 126 MHz): 20.4, 29.1, 30.3, 36.1, 40.8, 67.4, 75.4, 115.4, 115.5, 120.3, 120.8, 132.7, 142.6, 144.1, 146.0, 165.5, 173.1. [α]*_D_* (*c* = 1.0, CHCl_3_): −75. Lit. [16]: [α]*_D_* (*c* = 0.6, CHCl_3_): −76.

*(R)-1-((S)-6-oxo-3,6-Dihydro-2H-pyran-2-yl)propan-2-yl (E)-3-(3,4-dihydroxyphenyl)acrylate* (**4**). To a solution of EDC•HCl (143 mg, 0.75 mmol, 2 eq.) and DMAP (50 mg, 0.38 mmol, 1 eq.) in anhydrous CH_2_Cl_2_ (5 mL) were added a solution of acid **ix** (310mg, 0.75 mmol, 2 eq.) and alcohol 5 (60 mg, 0.31 mmol, 1 eq.) in anhydrous CH_2_Cl_2_ (5 mL) at 25 °C, and the mixture was stirred at 25 °C for 1 day. Upon completion, the reaction contents were diluted in EtOAc (90 mL), and extracted with solution of HCl 0.5 M (40 mL). The organic phase was washed with aqueous solution of saturated NaHCO_3_ (30 mL), dried (MgSO_4_), and concentrated. The crude was dissolved in anhydrous THF (10 mL), and benzoic acid (231 mg, 1.9 mmol, 5 eq.) and solution of TBAF 1 M in THF (1.9 mL, 1.9 mmol, 5 eq.) were added at 0 °C. The reaction was stirred at this temperature for 60 min, then aqueous solution of saturated NaHCO_3_ (30 mL) was added, and the aqueous phase was extracted with EtOAc (2 × 60 mL). The organic phases were grouped, washed with brine (30 mL) and dried (MgSO_4_). The product was purified by column chromatography (SiO_2_, hexanes/EtOAc 60:40 to 30:70) to afford **4** (61 mg, 50%). Clear yellow oil. R*_f_* 0.50 (SiO_2_, hexanes/EtOAc 30:70). ^1^H-NMR δ (methanol-*d*_4_, 250 MHz): 1.35 (d, *J* = 6.3 Hz, 3H), 1.88–2.01 (m, 1H), 2.15–2.26 (m, 1H), 2.29–2.58 (m, 2H), 4.54–4.65 (m, 1H), 5.21 (st, *J* = 9.8 Hz, 1H), 5.96 (ddd, *J* = 9.8, 2.4, 0.8 Hz, 1H), 6.24 (d, *J* = 15.8 Hz, 1H), 6.78 (d, *J* = 8.2 Hz, 1H), 6.94 (dd, *J* = 8.2, 1.9 Hz, 1H), 6.97–7.03 (m, 1H), 7.04 (d, *J* = 2.0 Hz, 1H), 7,54 (d, *J* = 15.9 Hz, 1H). ^13^C-NMR δ (methanol-*d*_4_, 62.9 MHz): 20.5, 30.3, 41.9, 68.8, 77.1, 115.3, 115.5, 116.6, 121.5, 123.1, 127.9, 146.9, 147.1, 148.2, 149.7, 166.7, 168.8. [α]*_D_* (*c* = 1.0, MeOH): −79. IR (NaCl, cm^−1^): 813, 1180, 1262, 1633, 1694, 2934, 2978, 3445 (broad).

### 3.2. Biological Experiments

All experimental procedures were performed in accordance with the guidelines of the Brazilian College for Animal Experimentation and were approved by the Ethics Committee at the University of Campinas. Six-week old male Swiss albinus mice were obtained from the University of Campinas Animal Breeding Center and maintained in individual cages at 21 ± 2 °C, with a 12/12 h dark-light cycle with food and water *ad libitum*. Mice were fed on a high fat diet containing 31% (w/w) of fat from lard.

After 6 h of fasting (starting 0800 h), mice were divided into three groups treated by an intraperitoneal (ip) injection with a single dose of either tarchonanthuslactone (3.0 mg/kg), dihydrocaffeic acid (3.0 mg/kg) or caffeic acid (3.0 mg/kg). Then, blood glucose levels were measured after 30, 60 and 90 min.

Long-term and cumulative effects of tarchonanthuslactone were evaluated in Swiss mice fed on high fat diet for eight weeks and treated three times a week, for four weeks with 3.0 mg/kg per dose ip of tarchonanthuslactone. Two days after the last dose of the compound, the mice were submitted to a 6-hour fasting and blood glucose levels were determined and expressed as percentage of saline treated group. Furthermore, lean non-diabetic Swiss mice fed on a commercial chow diet with approximately 4% (w/w) of fat were treated by an intraperitoneal injection with a single dose of tarchonanthuslactone (3.0 mg/kg) and the control group received saline. Blood glucose levels were measured after 30, 60 and 90 min and the area under this curve was calculated and expressed as percentage of saline treated group.

The number of animals in each group was at least six. Single blood samples were obtained from the tip of the tail and glucose levels were immediately measured using a glucometer from Abbott (Opptimum, Abbott Diabetes Care Inc., Alameda, CA, USA).

## Supplementary Materials

Supplementary materials can be accessed at: http://www.mdpi.com/1420-3049/20/03/5038/s1.
