# Supplementary material for: (−)-Tarchonanthuslactone Exerts a Blood Glucose-Increasing Effect in Experimental Type 2 Diabetes Mellitus
Source: Molecules. 2015 Mar 19;20(3):5038–49. doi: 10.3390/molecules20035038 (PMC6272554; doi:10.3390/molecules20035038)
Supplement: Supplementary file 1 [file molecules-20-05038-s001.pdf]

Supplementary Materials

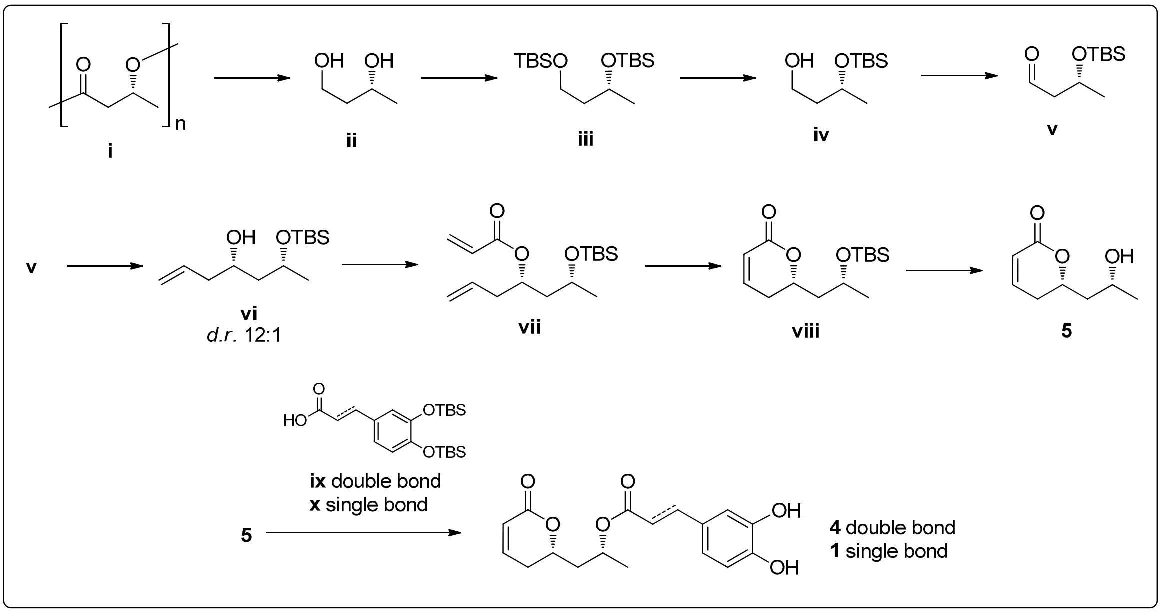

Scheme S1. Synthesis of tarchonanthuslactone **1** and analogue **4**.

**Acquisition Time (sec)**	3.1654	**Comment**	LFTN 77 CDCL3 250MHz			**Date**	09 Aug 2012 17:07:28
**Date Stamp**	09 Aug 2012 17:07:28						
**File Name**	C:\Users\Luiz\Desktop\Luiz\Lab Pilli\Experimentais\Análises (CG, RMN, IV, HPLC)\RMN\LFTN77 - ago09cmaH1\1\pdata\1\1r						
**Frequency (MHz)**	250.13	**Nucleus**	1H	**Number of Transients**	11	**Origin**	spect
**Original Points Count**	16384	**Owner**	root	**Points Count**	32768	**Pulse Sequence**	zg30
**Receiver Gain**	128.00	**SW(cyclical) (Hz)**	5175.98	**Solvent**	CHLOROFORM-d	**Spectrum Offset (Hz)**	1750.9441
**Spectrum Type**	STANDARD	**Sweep Width (Hz)**	5175.83	**Temperature (degree C)**	25.160		

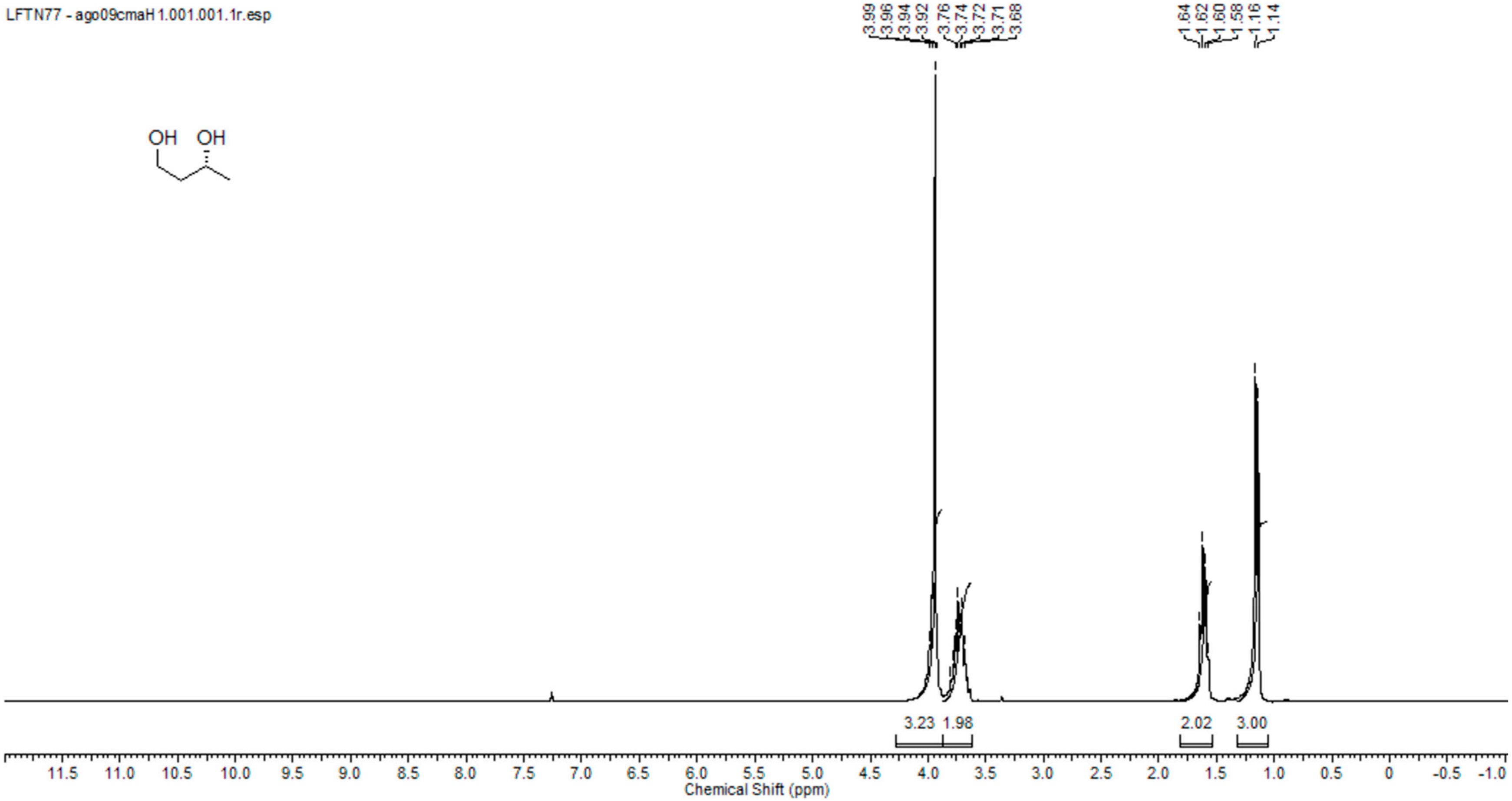

Figure S1. ^{1}H-NMR (250 MHz, $CDCl_3$) of compound **ii**.

**Acquisition Time (sec)**	0.5439	**Comment**	LFTN 77 CDCL3 250MHz			**Date**	09 Aug 2012 17:26:40
**Date Stamp**	09 Aug 2012 17:26:40						
**File Name**	C:\Users\Luiz\Desktop\Luiz\Lab Pilli\Experimentais\Análises (CG, RMN, IV, HPLC)\RMN\LFTN77- ago09cmaC\1\pdata\1\1r						
**Frequency (MHz)**	62.90	**Nucleus**	13C	**Number of Transients**	386	**Origin**	spect
**Original Points Count**	8192	**Owner**	root	**Points Count**	32768	**Pulse Sequence**	zgpg30
**Receiver Gain**	362.00	**SW(cyclical) (Hz)**	15060.24	**Solvent**	CHLOROFORM-d	**Spectrum Offset (Hz)**	6295.4580
**Spectrum Type**	STANDARD	**Sweep Width (Hz)**	15059.78	**Temperature (degree C)**	25.160		

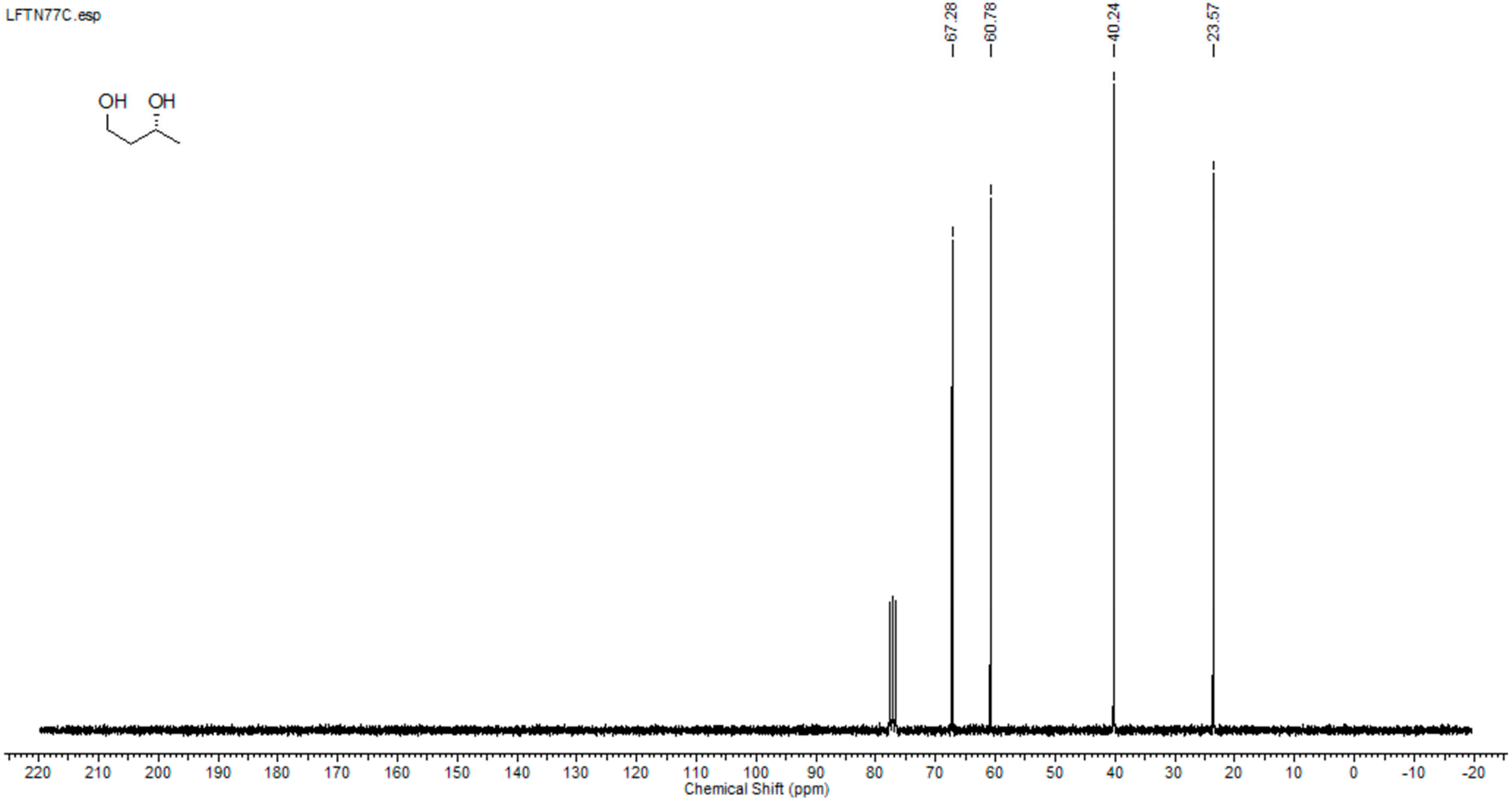

Figure S2. ^{13}C-NMR (62.9 MHz, $CDCl_3$) of compound **ii**.

Acquisition Time (sec)	3.1654	***Comment***	LFTN83 CDCL3 250MHz			***Date***	22 Aug 2012 09:13:52
Date Stamp	22 Aug 2012 09:13:52						
File Name	C:\Users\Luiz\Desktop\Luiz\Lab Pilli\Experimentais\Análises (CG, RMN, IV, HPLC)\RMN\LFTN83 - ago22cmaH1\1\pdata\1\1r						
Frequency (MHz)	250.13	***Nucleus***	1H	***Number of Transients***	16	***Origin***	spect
Original Points Count	16384	***Owner***	root	***Points Count***	32768	***Pulse Sequence***	zg30
Receiver Gain	101.60	***SW(cyclical) (Hz)***	5175.98	***Solvent***	CHLOROFORM-d	***Spectrum Offset (Hz)***	1750.6281
Spectrum Type	STANDARD	***Sweep Width (Hz)***	5175.83	***Temperature (degree C)***	25.160		

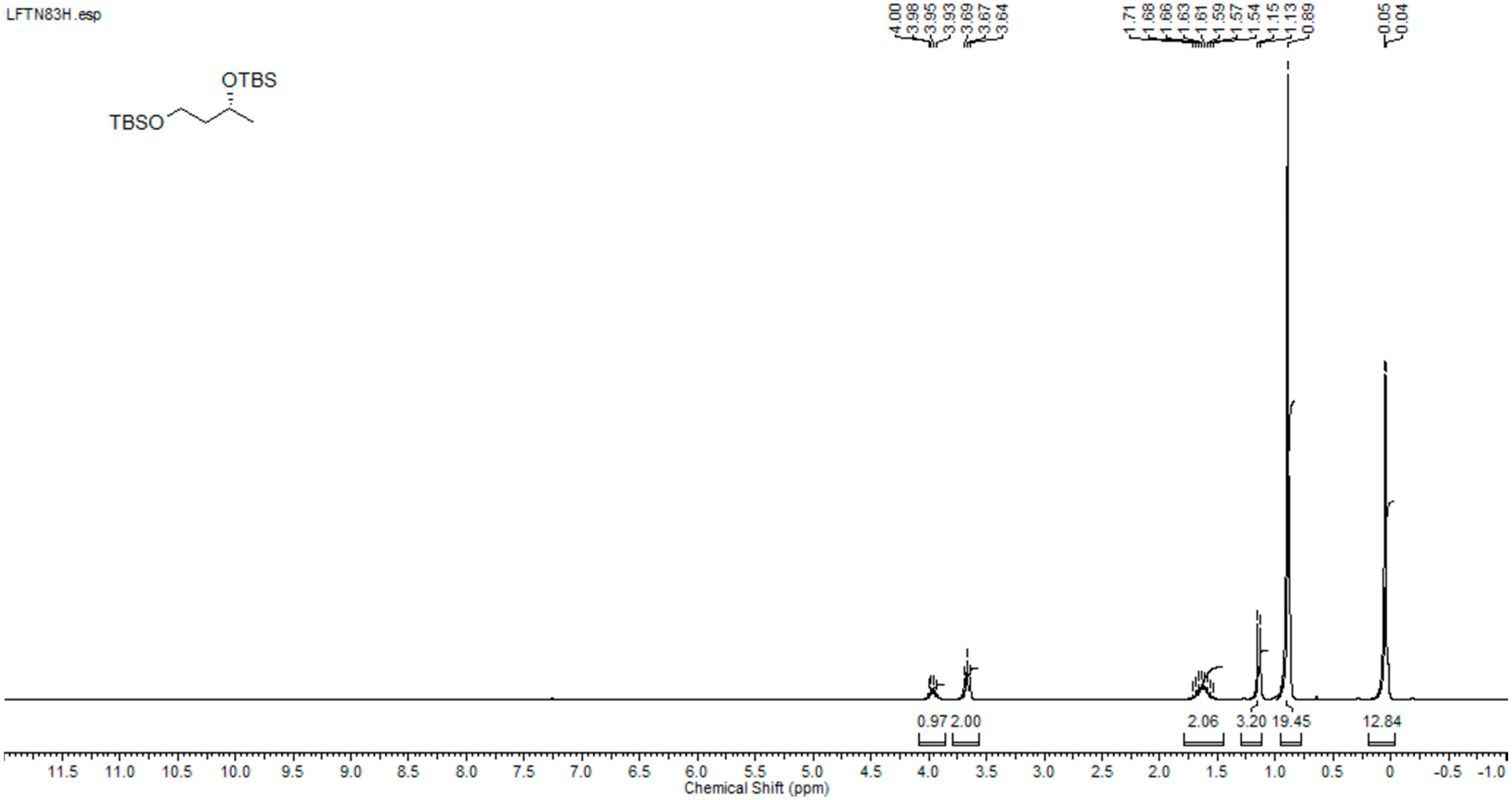

Figure S3. ^{1}H-NMR (250 MHz, $CDCl_3$) of compound **iii**.

Acquisition Time (sec)	0.5439	*Comment*	LFTN83 CDCL3 250MHz C13			*Date*	22 Aug 2012 10:07:12
Date Stamp	22 Aug 2012 10:07:12						
File Name	C:\Users\Luiz\Desktop\Luiz\Lab Pilli\Experimentais\Análises (CG, RMN, IV, HPLC)\RMN\LFTN83 - ago22cmaC\2\pdata\1\1r						
Frequency (MHz)	62.90	*Nucleus*	13C	*Number of Transients*	847	*Origin*	spect
Original Points Count	8192	*Owner*	root	*Points Count*	32768	*Pulse Sequence*	zgpg30
Receiver Gain	456.10	*SW(cyclical) (Hz)*	15060.24	*Solvent*	CHLOROFORM-d	*Spectrum Offset (Hz)*	6303.2710
Spectrum Type	STANDARD	*Sweep Width (Hz)*	15059.78	*Temperature (degree C)*	25.160		

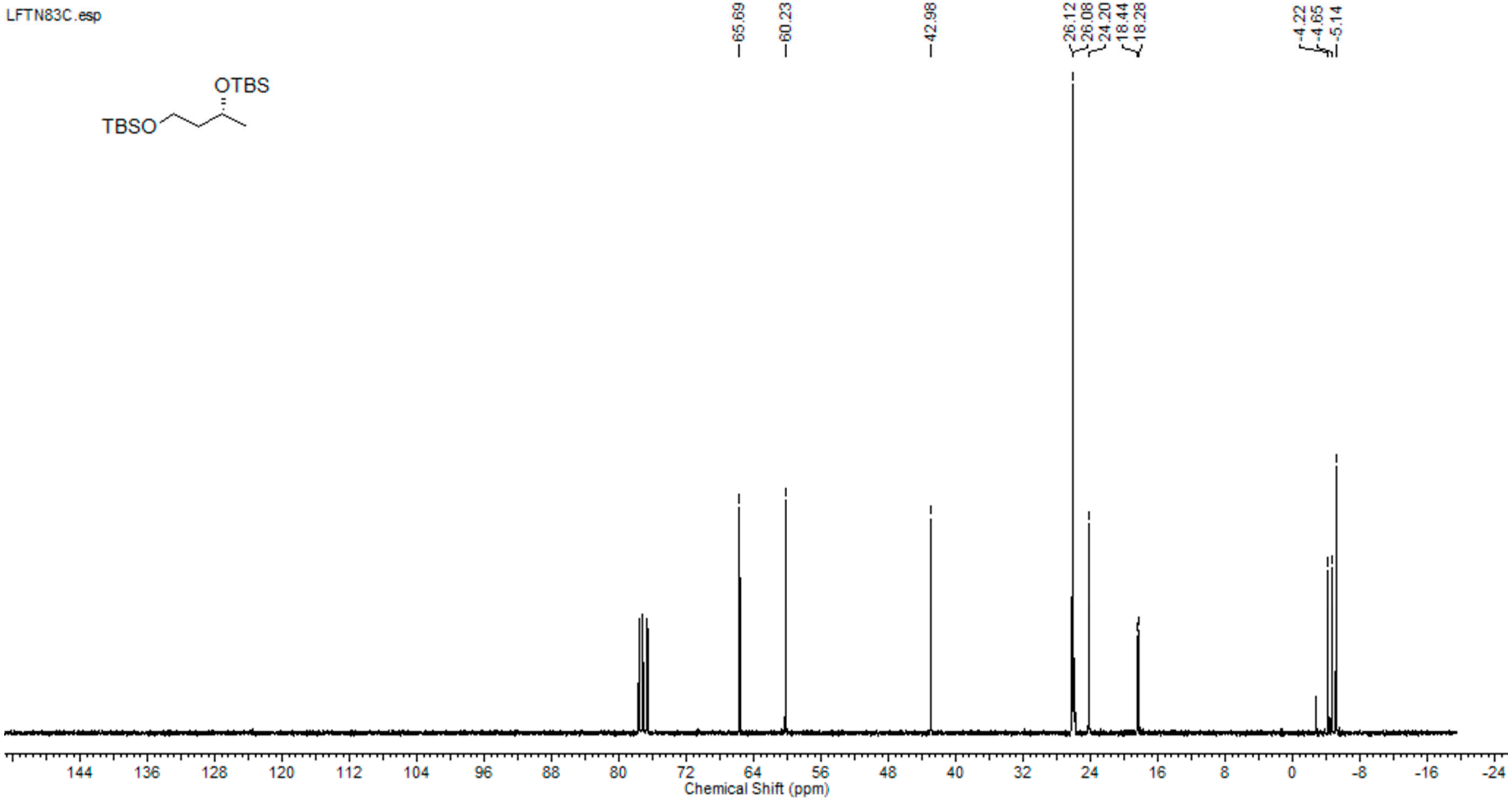

Figure S4. ^{13}C-NMR (62.9 MHz, $CDCl_3$) of compound **iii**.

Acquisition Time (sec)	3.1654	Comment	LFTN85 CDCL3 250MHz H1			Date	29 Aug 2012 11:13:20
Date Stamp	29 Aug 2012 11:13:20						
File Name	C:\Users\Luiz\Desktop\Luiz\Lab Pilli\Experimentais\Análises (CG, RMN, IV, HPLC)\RMN\LFTN85 - ago29cmaH6\1\pdata\1\1r						
Frequency (MHz)	250.13	Nucleus	1H	Number of Transients	1	Origin	spect
Original Points Count	16384	Owner	root	Points Count	32768	Pulse Sequence	zg30
Receiver Gain	128.00	SW(cyclical) (Hz)	5175.98	Solvent	CHLOROFORM-d	Spectrum Offset (Hz)	1750.9441
Spectrum Type	STANDARD	Sweep Width (Hz)	5175.83	Temperature (degree C)	25.260		

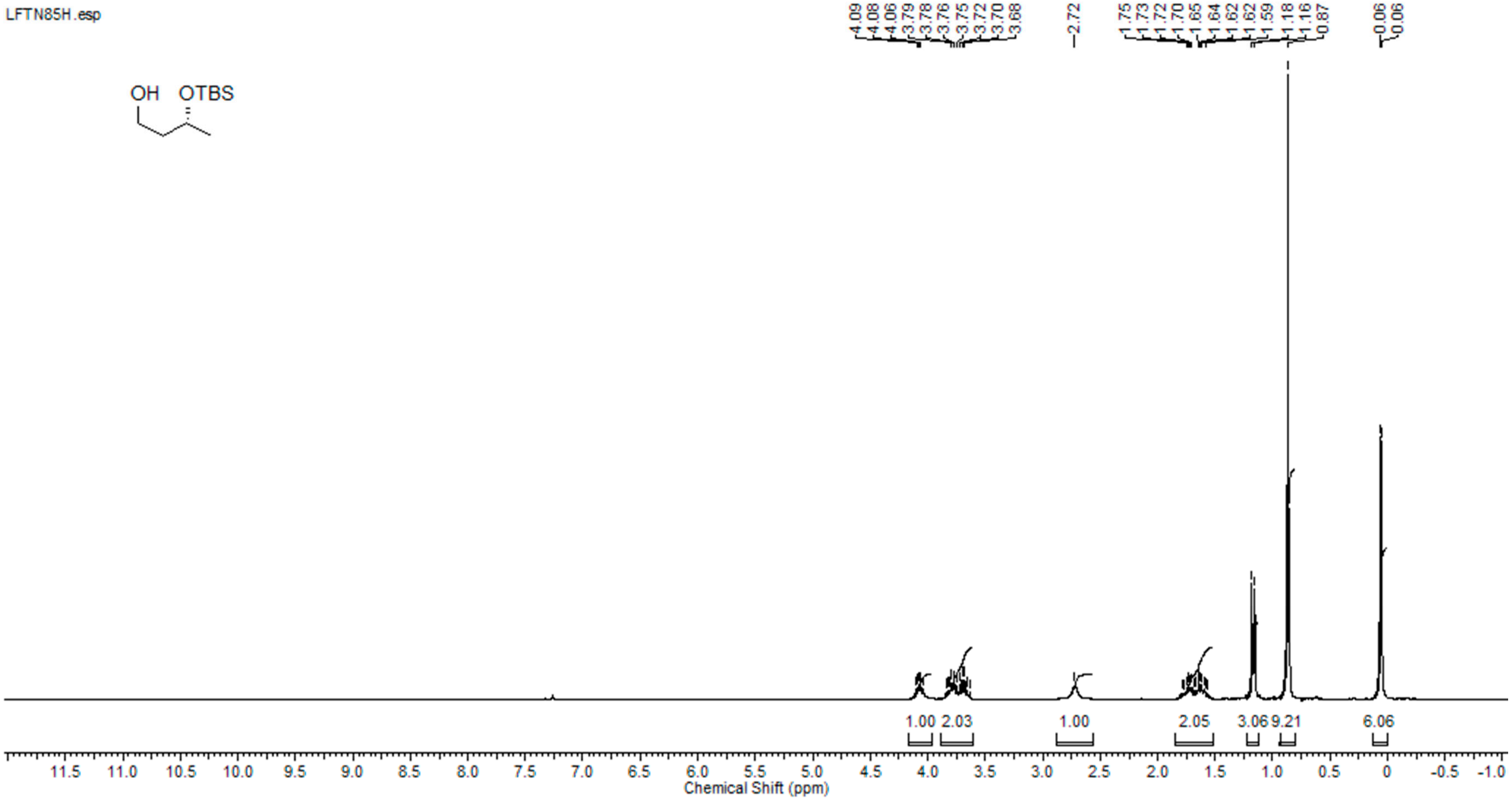

Figure S5. ^{1}H-NMR (250 MHz, $CDCl_3$) of compound **iv**.

Acquisition Time (sec)	0.5439	Comment	LFTN85 CDCL3 250MHz C13			Date	29 Aug 2012 11:43:12
Date Stamp	29 Aug 2012 11:43:12						
File Name	C:\Users\Luiz\Desktop\Luiz\Lab Pilli\Experimentais\Análises (CG, RMN, IV, HPLC)\RMN\LFTN85 - ago29cmaH6\3\pdata\1\1r						
Frequency (MHz)	62.90	Nucleus	13C	Number of Transients	538	Origin	spect
Original Points Count	8192	Owner	root	Points Count	32768	Pulse Sequence	zgpg30
Receiver Gain	512.00	SW(cyclical) (Hz)	15060.24	Solvent	CHLOROFORM-d	Spectrum Offset (Hz)	6299.1348
Spectrum Type	STANDARD	Sweep Width (Hz)	15059.78	Temperature (degree C)	25.160		

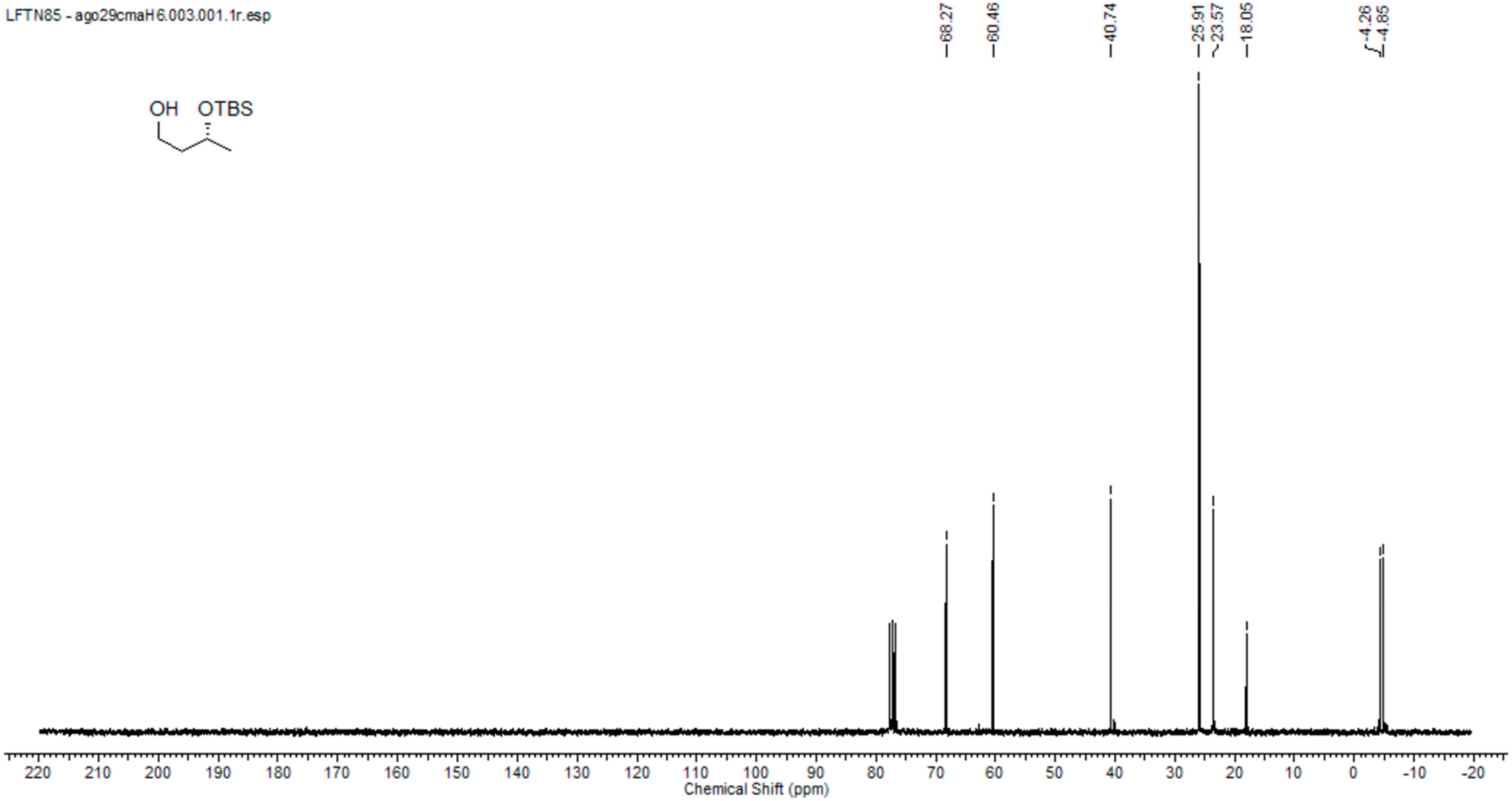

Figure S6. ^{13}C-NMR (62.9 MHz, $CDCl_3$) of compound **iv**.

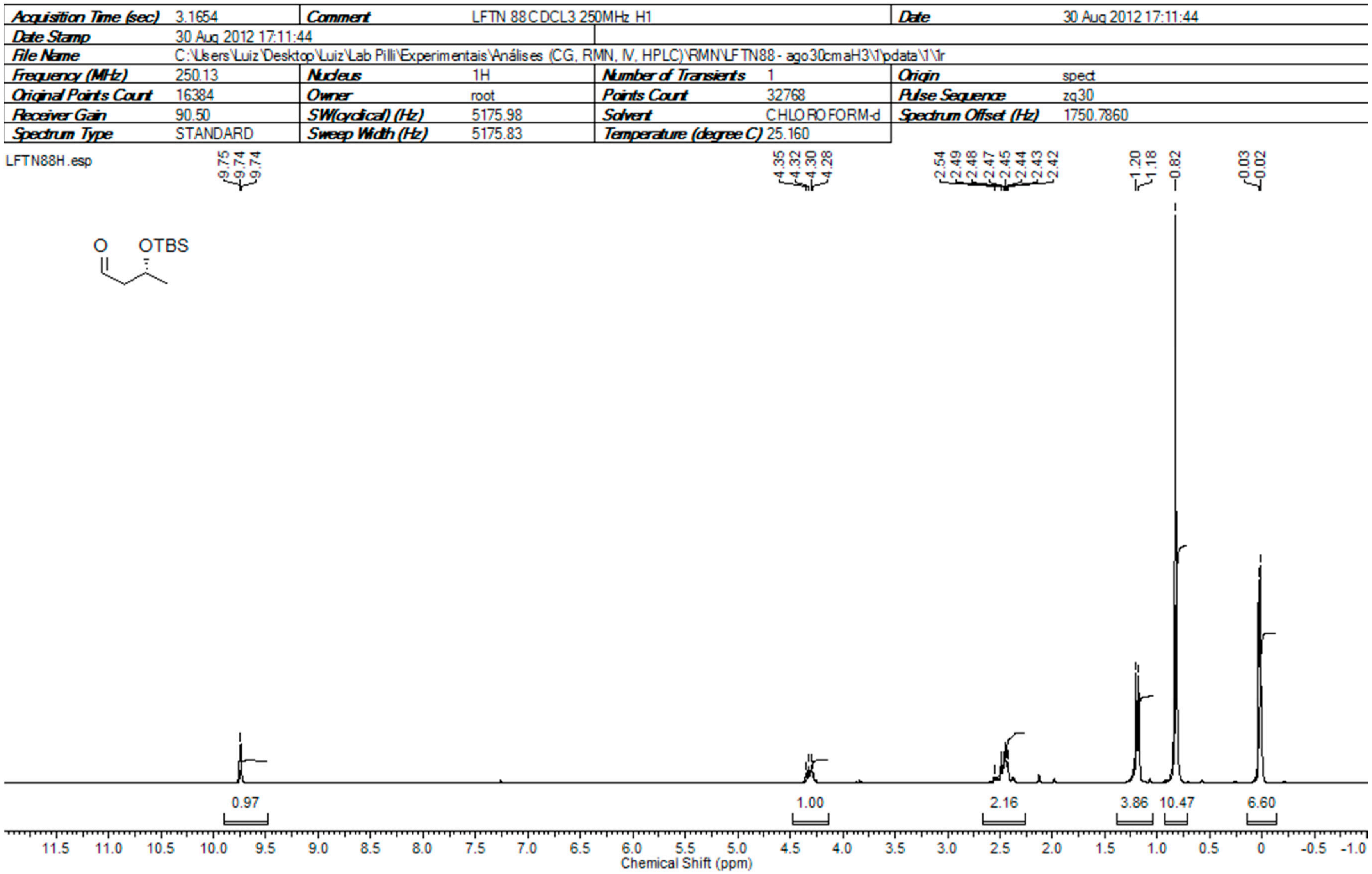

Figure S7. ^{1}H-NMR (250 MHz, $CDCl_3$) of compound **v**.

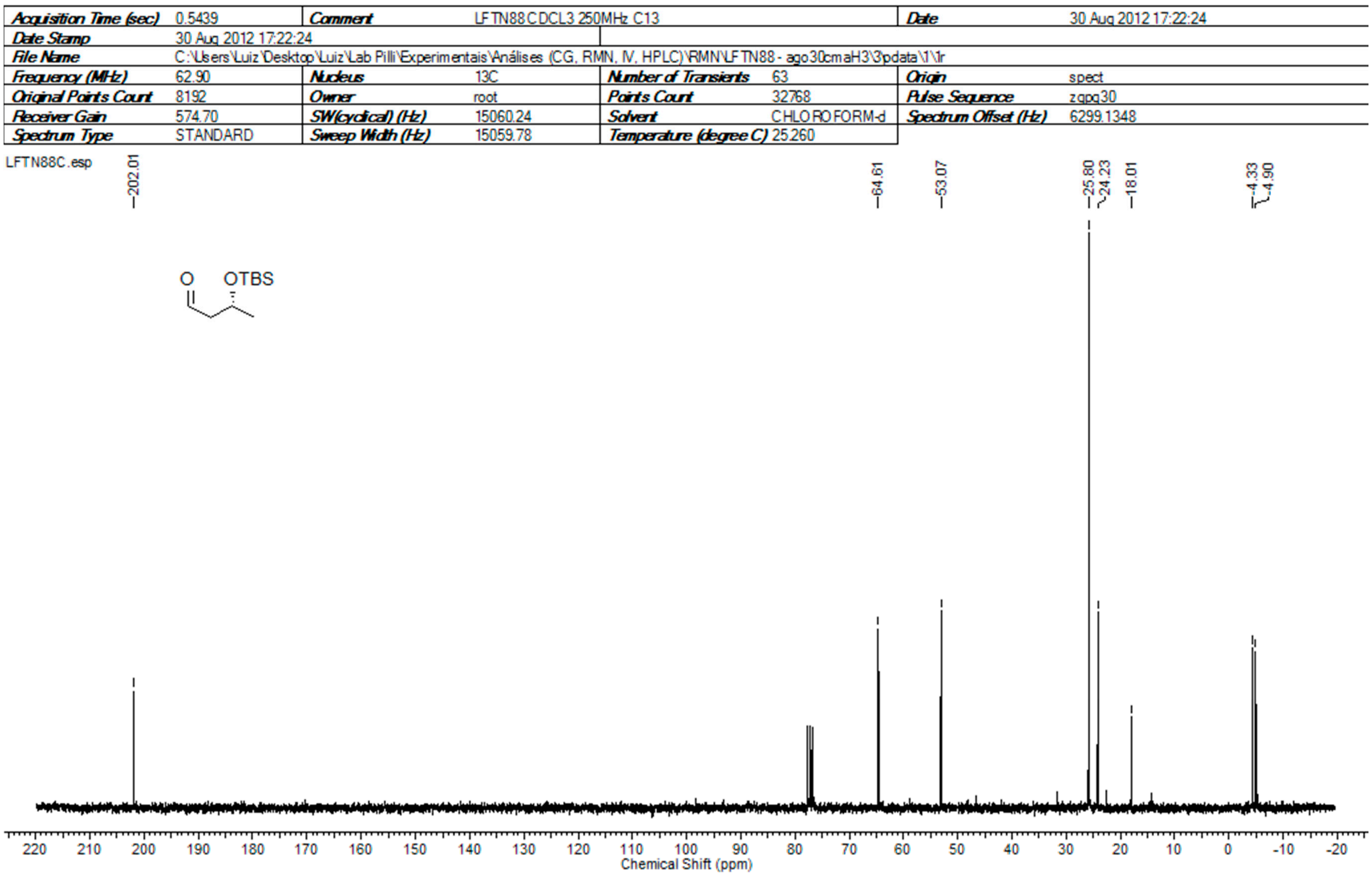

Figure S8. ^{13}C-NMR (62.9 MHz, $CDCl_3$) of compound **v**.

Acquisition Time (sec)	3.1654	***Comment***	LFTN91 CDCL3 250MHz CDCL3			***Date***	06 Sep 2012 17:07:28
Date Stamp	06 Sep 2012 17:07:28						
File Name	C:\Users\Luiz\Desktop\Luiz\Lab Pilli\Experimentais\Análises (CG, RMN, IV, HPLC)\RMN\LFTN91 - set06cmaH2\1\pdata\1\1r						
Frequency (MHz)	250.13	***Nucleus***	1H	***Number of Transients***	11	***Origin***	spect
Original Points Count	16384	***Owner***	root	***Points Count***	32768	***Pulse Sequence***	zg30
Receiver Gain	101.60	***SW(cyclical) (Hz)***	5175.98	***Solvent***	CHLOROFORM-d	***Spectrum Offset (Hz)***	1750.4702
Spectrum Type	STANDARD	***Sweep Width (Hz)***	5175.83	***Temperature (degree C)***	25.160		

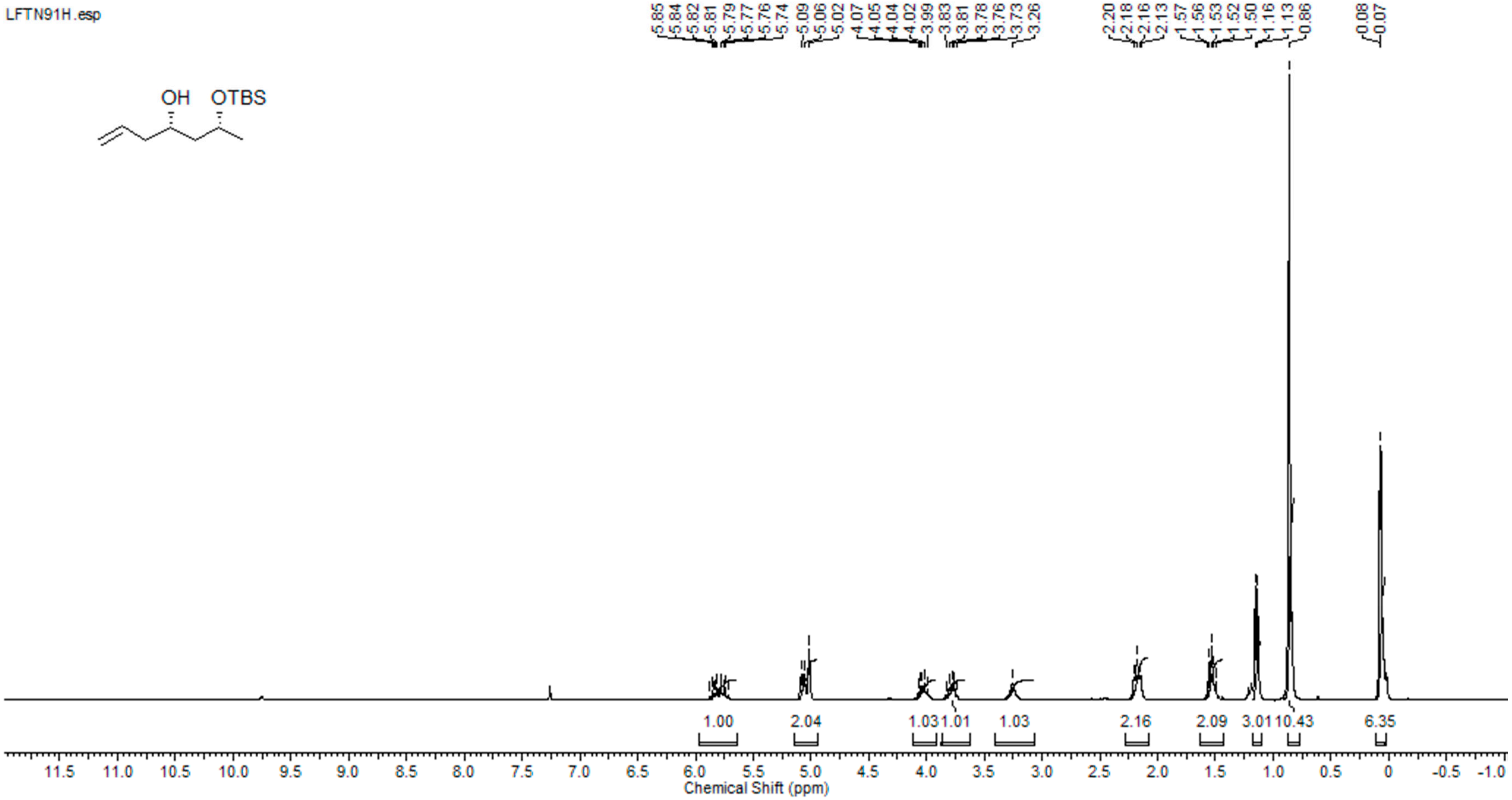

Figure S9. ^{1}H-NMR (250 MHz, $CDCl_3$) of compound **vi**.

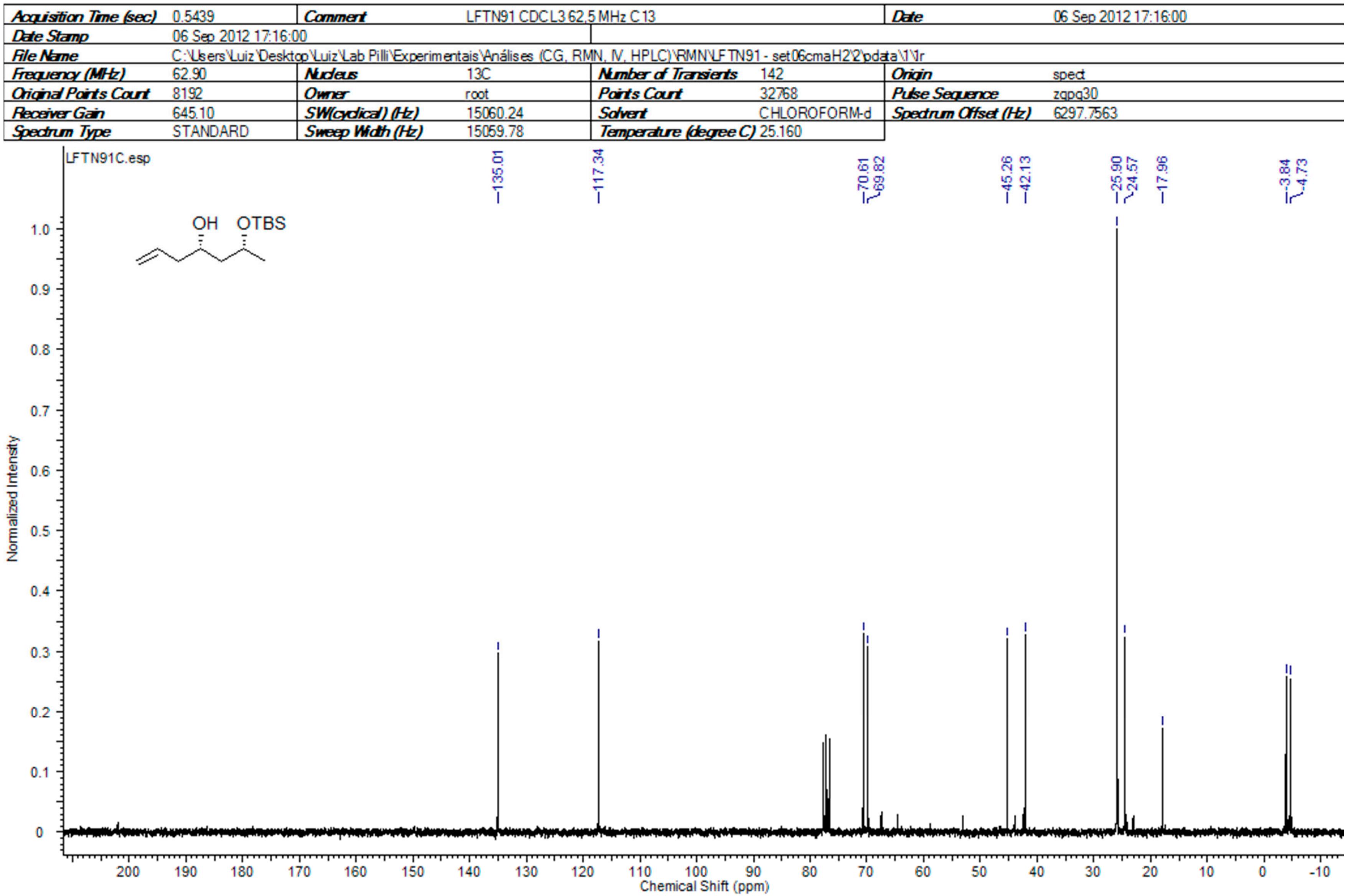

Figure S10. ^{13}C-NMR (62.9 MHz, $CDCl_3$) of compound **vi**.

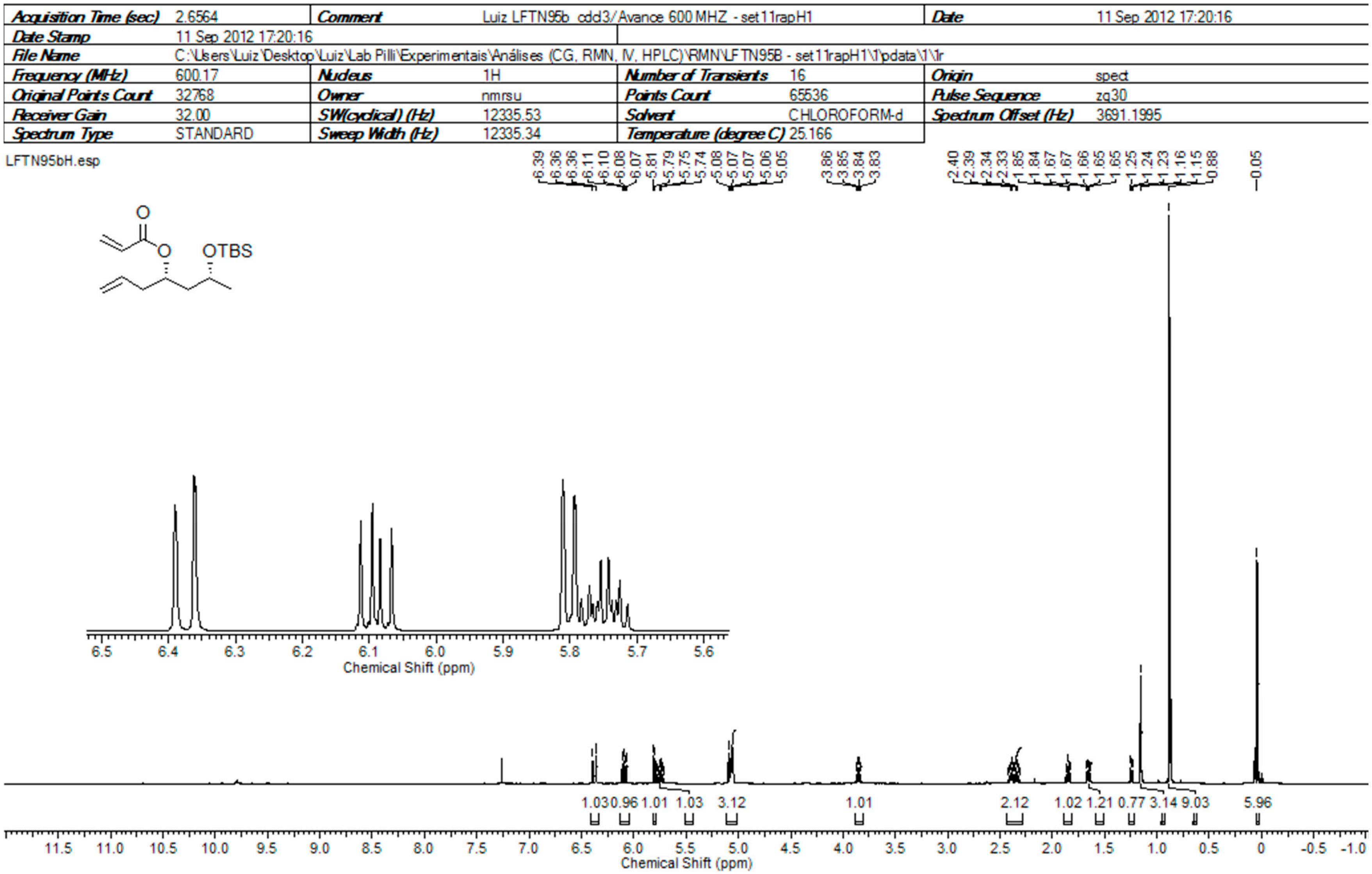

Figure S11. ^{1}H-NMR (600 MHz, $CDCl_3$) of compound **vii**.

**Acquisition Time (sec)**	0.4544	**Comment**	Luiz LFTN95b cdcl3/Avance 600 MHZ - set11rapC1			**Date**	11 Sep 2012 08:03:28
**Date Stamp**	11 Sep 2012 08:03:28						
**File Name**	C:\Users\Luiz\Desktop\Luiz\Lab Pilli\Experimentais\Análises (CG, RMN, IV, HPLC)\RMN\LFTN95B - set11rapC1\1\pdata\1\1r						
**Frequency (MHz)**	150.91	**Nucleus**	13C	**Number of Transients**	128	**Origin**	spect
**Original Points Count**	16384	**Owner**	nmrsu	**Points Count**	32768	**Pulse Sequence**	zgpg30
**Receiver Gain**	203.00	**SW(cyclical) (Hz)**	36057.69	**Solvent**	CHLOROFORM-d	**Spectrum Offset (Hz)**	15109.0264
**Spectrum Type**	STANDARD	**Sweep Width (Hz)**	36056.59	**Temperature (degree C)**	25.354		

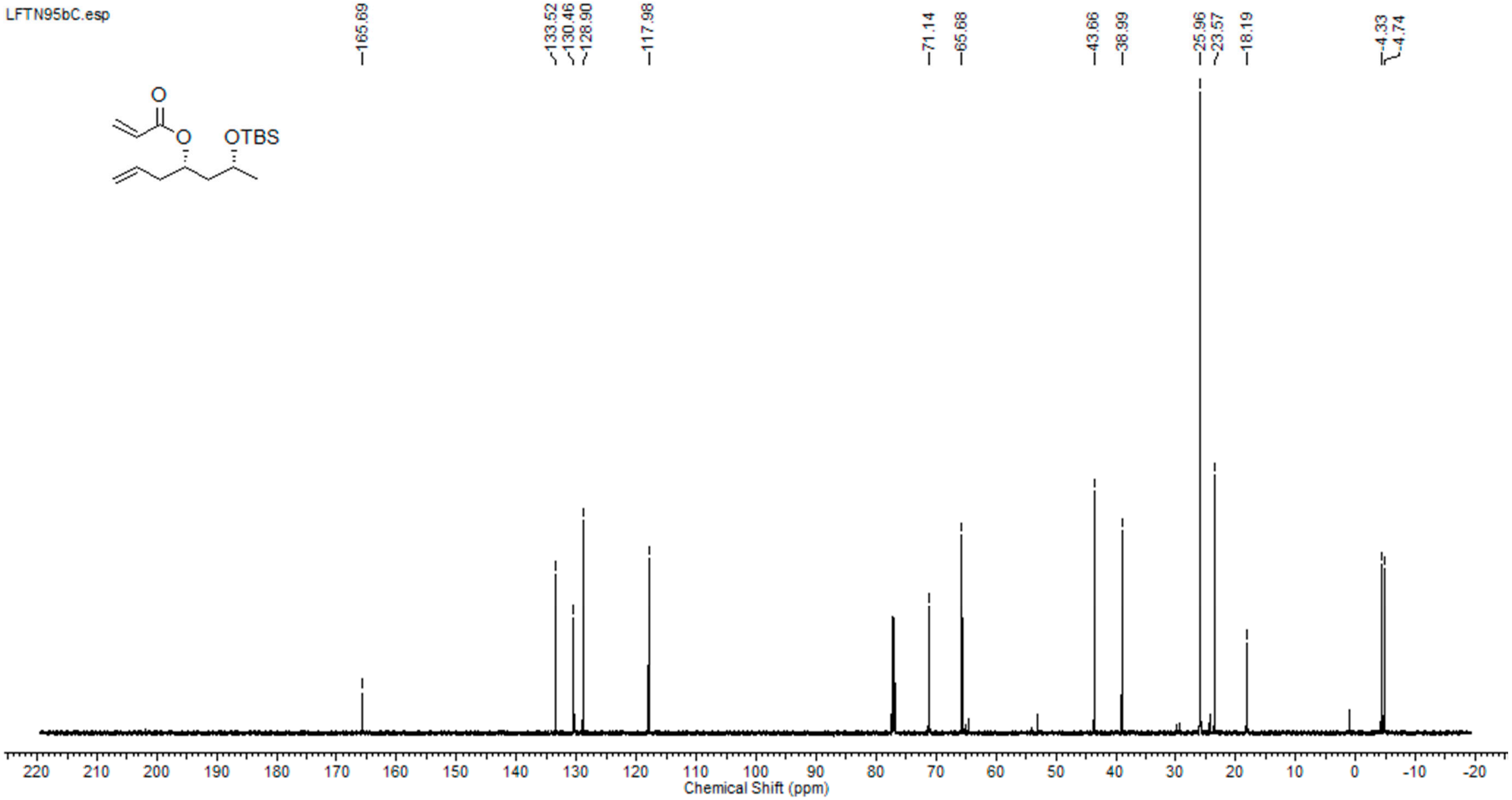

Figure S12. ^{13}C-NMR (150 MHz, $CDCl_3$) of compound **vii**.

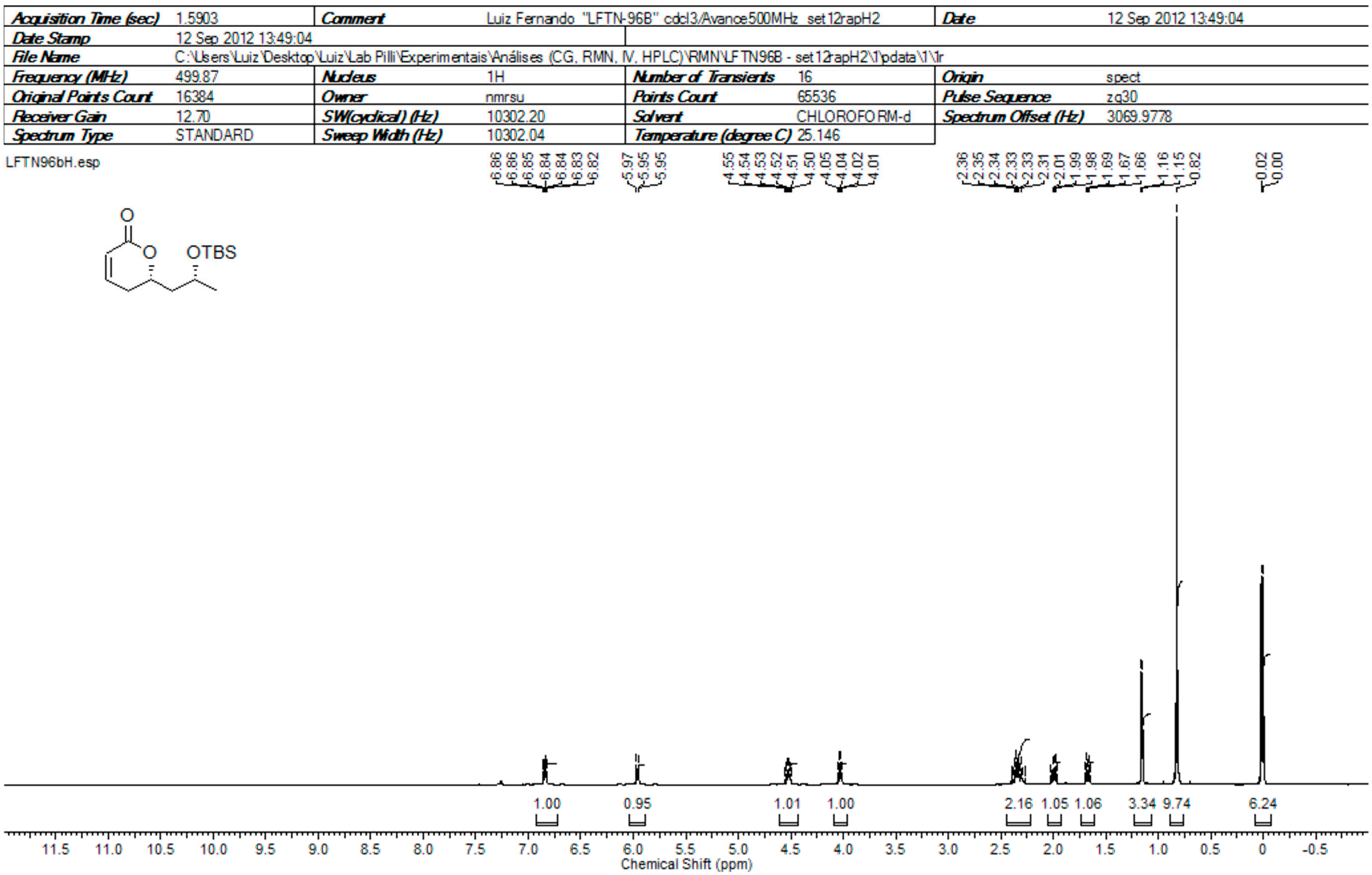

Figure S13. ^{1}H-NMR (500 MHz, $CDCl_3$) of compound **viii**.

**Acquisition Time (sec)**	0.4981	**Comment**	Luiz Fernando "LFTN-96B" cdcl3/Avance500MHz set12rapC2			**Date**	12 Sep 2012 14:08:16
**Date Stamp**	12 Sep 2012 14:08:16						
**File Name**	C:\Users\Luiz\Desktop\Luiz\Lab Pilli\Experimentais\Análises (CG, RMN, IV, HPLC)\RMN\LFTN96B - set12rapH2\2\pdata\1\1r						
**Frequency (MHz)**	125.69	**Nucleus**	13C	**Number of Transients**	512	**Origin**	spect
**Original Points Count**	16384	**Owner**	nmrsu	**Points Count**	32768	**Pulse Sequence**	zgpg30
**Receiver Gain**	2050.00	**SW(cyclical) (Hz)**	32894.74	**Solvent**	CHLOROFORM-d	**Spectrum Offset (Hz)**	13829.8457
**Spectrum Type**	STANDARD	**Sweep Width (Hz)**	32893.73	**Temperature (degree C)**	25.150		

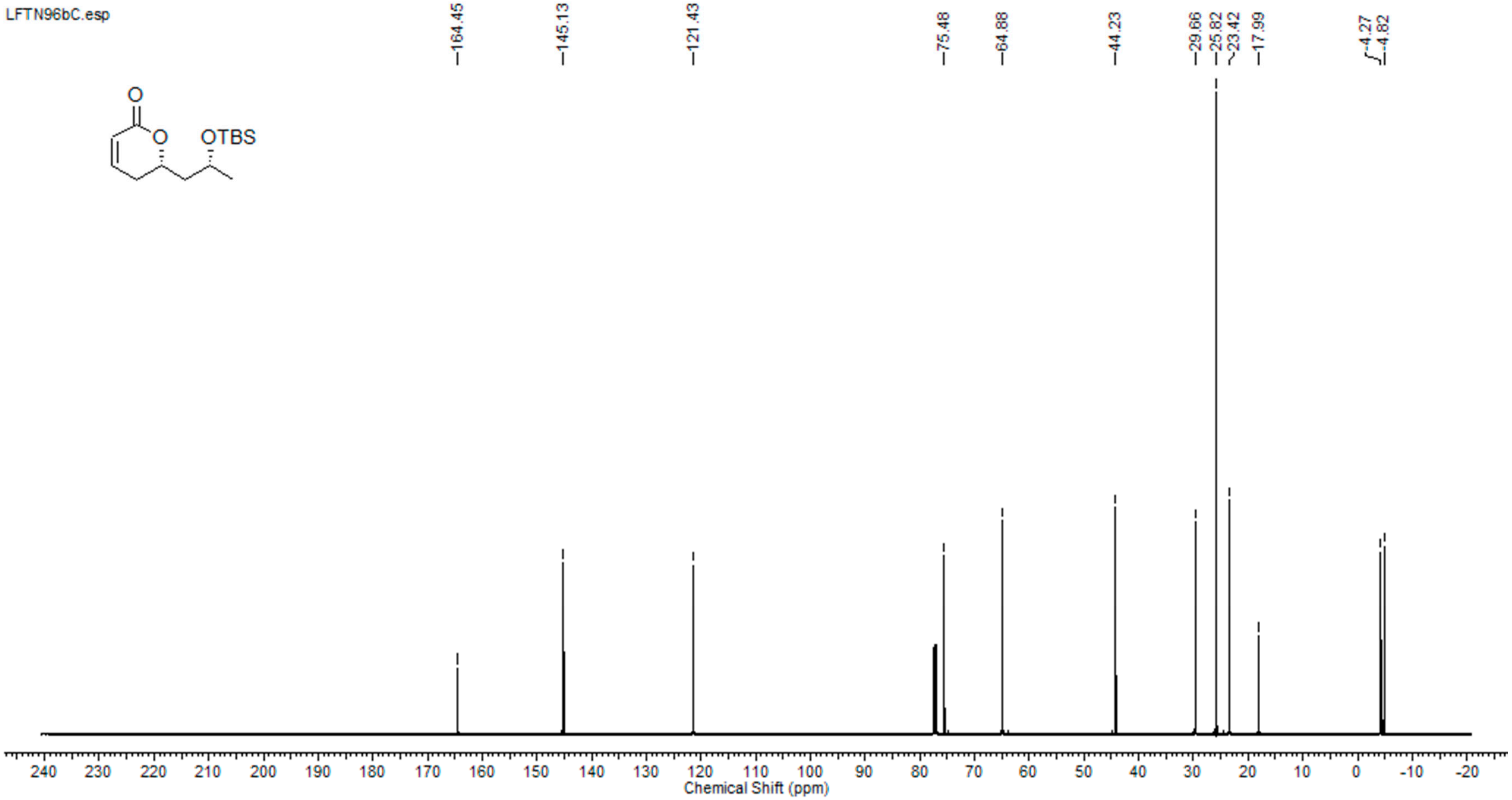

Figure S14. ^{13}C-NMR (125 MHz, $CDCl_3$) of compound **viii**.

Acquisition Time (sec)	1.6384	Comment	LFTN121 CDCL3 500MHz			Date	30 Oct 2012 21:45:04
Date Stamp	30 Oct 2012 21:45:04						
File Name	C:\Users\Luiz\Desktop\Luiz\Lab Pilli\Experimentais\Análises (CG, RMN, IV, HPLC)\RMN\LFTN121 - out30cmaH1\1\pdata\1\1r						
Frequency (MHz)	499.87	Nucleus	1H	Number of Transients	16	Origin	spect
Original Points Count	16384	Owner	nmrsu	Points Count	65536	Pulse Sequence	zg30
Receiver Gain	32.00	SW(cyclical) (Hz)	10000.00	Solvent	CHLOROFORM-d	Spectrum Offset (Hz)	3070.2031
Spectrum Type	STANDARD	Sweep Width (Hz)	9999.85	Temperature (degree C)	25.140		

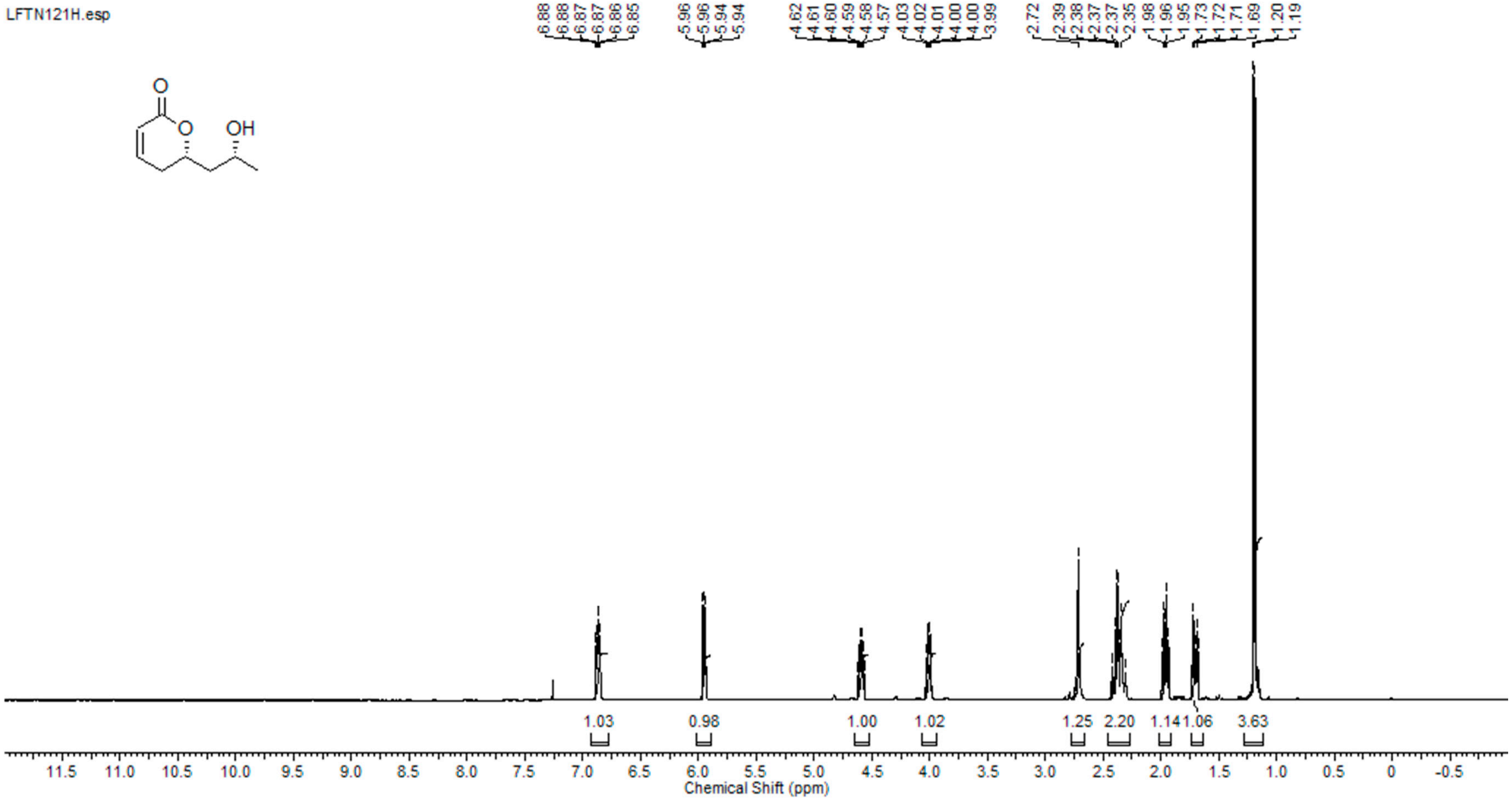

Figure S15. ^{1}H-NMR (500 MHz, $CDCl_3$) of compound **5**.

Acquisition Time (sec)	0.5505	*Comment*	LFTN121 CDCL3 500MHz C13			*Date*	30 Oct 2012 22:32:00
Date Stamp	30 Oct 2012 22:32:00						
File Name	C:\Users\Luiz\Desktop\Luiz\Lab Pilli\Experimentais\Análises (CG, RMN, IV, HPLC)\RMN\LFTN121 - out30cmaH1\2\pdata\1\1r						
Frequency (MHz)	125.69	*Nucleus*	13C	*Number of Transients*	1024	*Origin*	spect
Original Points Count	16384	*Owner*	nmrsu	*Points Count*	32768	*Pulse Sequence*	zgpg30
Receiver Gain	2050.00	*SW(cyclical) (Hz)*	29761.90	*Solvent*	CHLOROFORM-d	*Spectrum Offset (Hz)*	12568.9873
Spectrum Type	STANDARD	*Sweep Width (Hz)*	29761.00	*Temperature (degree C)*	25.143		

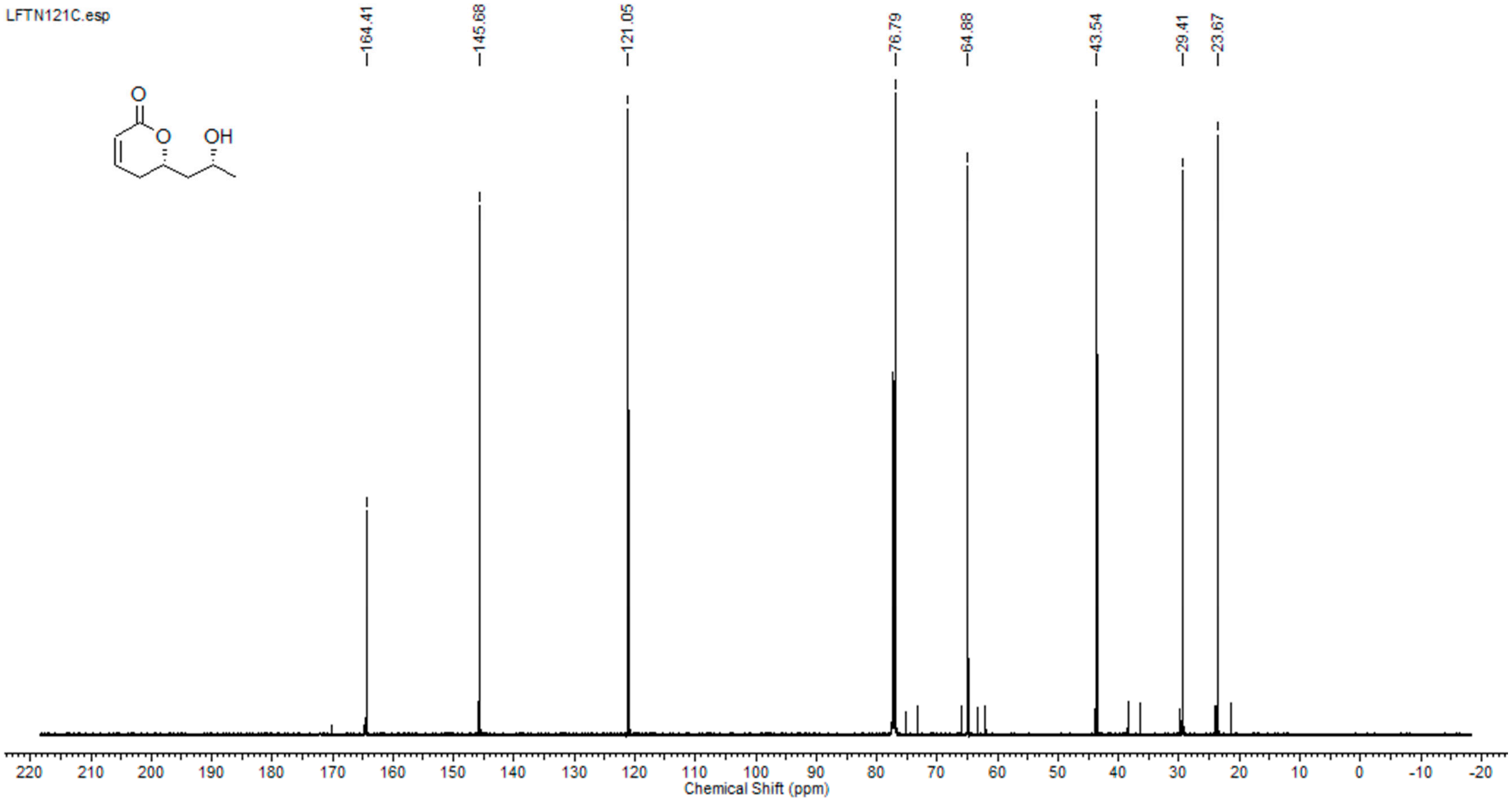

Figure S16. ^{13}C-NMR (125 MHz, $CDCl_3$) of compound **5**.

Acquisition Time (sec)	3.1654	Comment	Luiz - LFT010 - fev17rapH1			Date	17 Feb 2012 19:54:08
Date Stamp	17 Feb 2012 19:54:08						
File Name	C:\Users\Luiz\Desktop\Luiz\Lab Pilli\Experimentais\Análises (CG, RMN, IV, HPLC)\RMN\LFTN10 - ok - fev17rapH1\1\pdata\1\1r						
Frequency (MHz)	250.13	Nucleus	1H	Number of Transients	10	Origin	spect
Original Points Count	16384	Owner	root	Points Count	32768	Pulse Sequence	zg30
Receiver Gain	1625.50	SW(cyclical) (Hz)	5175.98	Solvent	CHLOROFORM-d	Spectrum Offset (Hz)	1750.4702
Spectrum Type	STANDARD	Sweep Width (Hz)	5175.83	Temperature (degree C)	26.260		

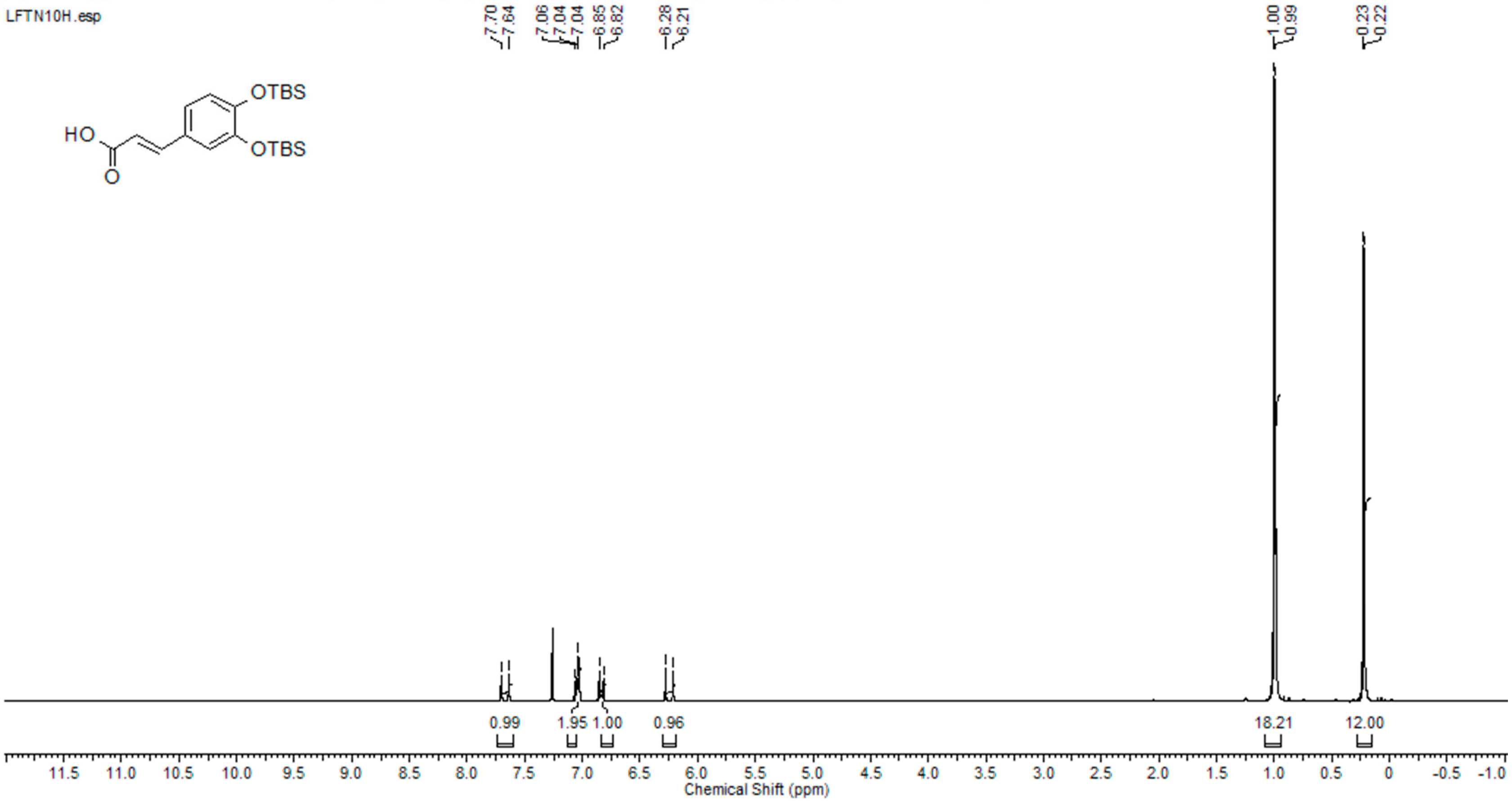

Figure S17. ^{1}H-NMR (250 MHz, $CDCl_3$) of compound **ix**.

Acquisition Time (sec)	0.5439	Comment	LFTN-10 CDCL3 250MHZ			Date	25 Feb 2012 15:48:32
Date Stamp	25 Feb 2012 15:48:32						
File Name	C:\Users\Luiz\Desktop\Luiz\Lab Pilli\Experimentais\Análises (CG, RMN, IV, HPLC)\RMN\LFTN10 - ok - fev25lftnC\1\pdata\1\1r						
Frequency (MHz)	62.90	Nucleus	13C	Number of Transients	1024	Origin	spect
Original Points Count	8192	Owner	root	Points Count	32768	Pulse Sequence	zgpg30
Receiver Gain	645.10	SW(cyclical) (Hz)	15060.24	Solvent	CHLOROFORM-d	Spectrum Offset (Hz)	6298.6753
Spectrum Type	STANDARD	Sweep Width (Hz)	15059.78	Temperature (degree C)	25.660		

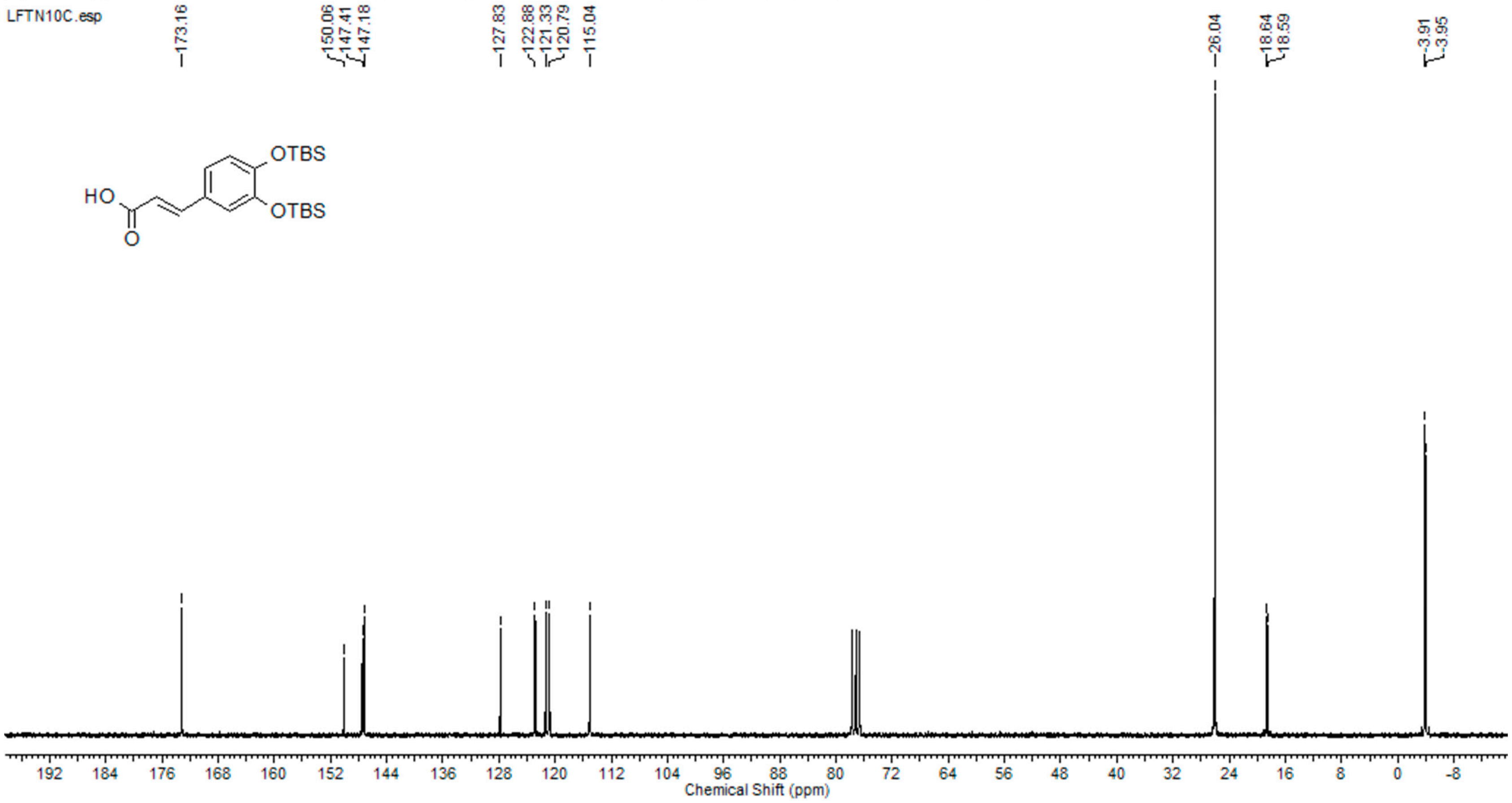

Figure S18. ^{13}C-NMR (62.9 MHz, $CDCl_3$) of compound **ix**.

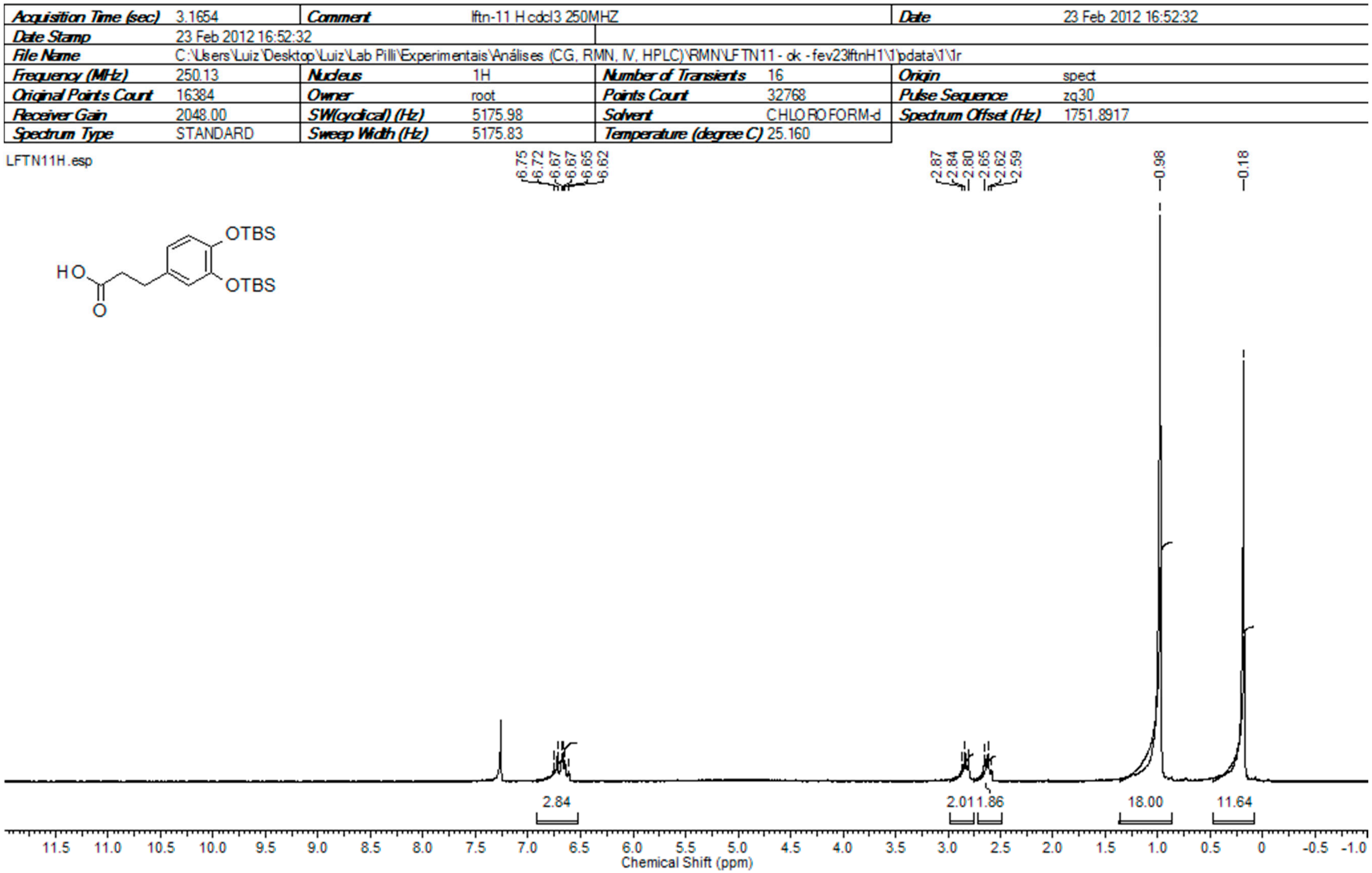

Figure S19. ^{1}H-NMR (250 MHz, $CDCl_3$) of compound **x**.

Acquisition Time (sec)	0.5439	***Comment***	LFTN-11C13 250MHZ CDCL3			***Date***	25 Feb 2012 17:13:52
Date Stamp	25 Feb 2012 17:13:52						
File Name	C:\Users\Luiz\Desktop\Luiz\Lab Pilli\Experimentais\Análises (CG, RMN, IV, HPLC)\RMN\LFTN11 - ok - fev25lftnC1\1\pdata\1\1r						
Frequency (MHz)	62.90	***Nucleus***	13C	***Number of Transients***	1024	***Origin***	spect
Original Points Count	8192	***Owner***	root	***Points Count***	32768	***Pulse Sequence***	zgpg30
Receiver Gain	574.70	***SW(cyclical) (Hz)***	15060.24	***Solvent***	CHLOROFORM-d	***Spectrum Offset (Hz)***	6296.8364
Spectrum Type	STANDARD	***Sweep Width (Hz)***	15059.78	***Temperature (degree C)***	25.860		

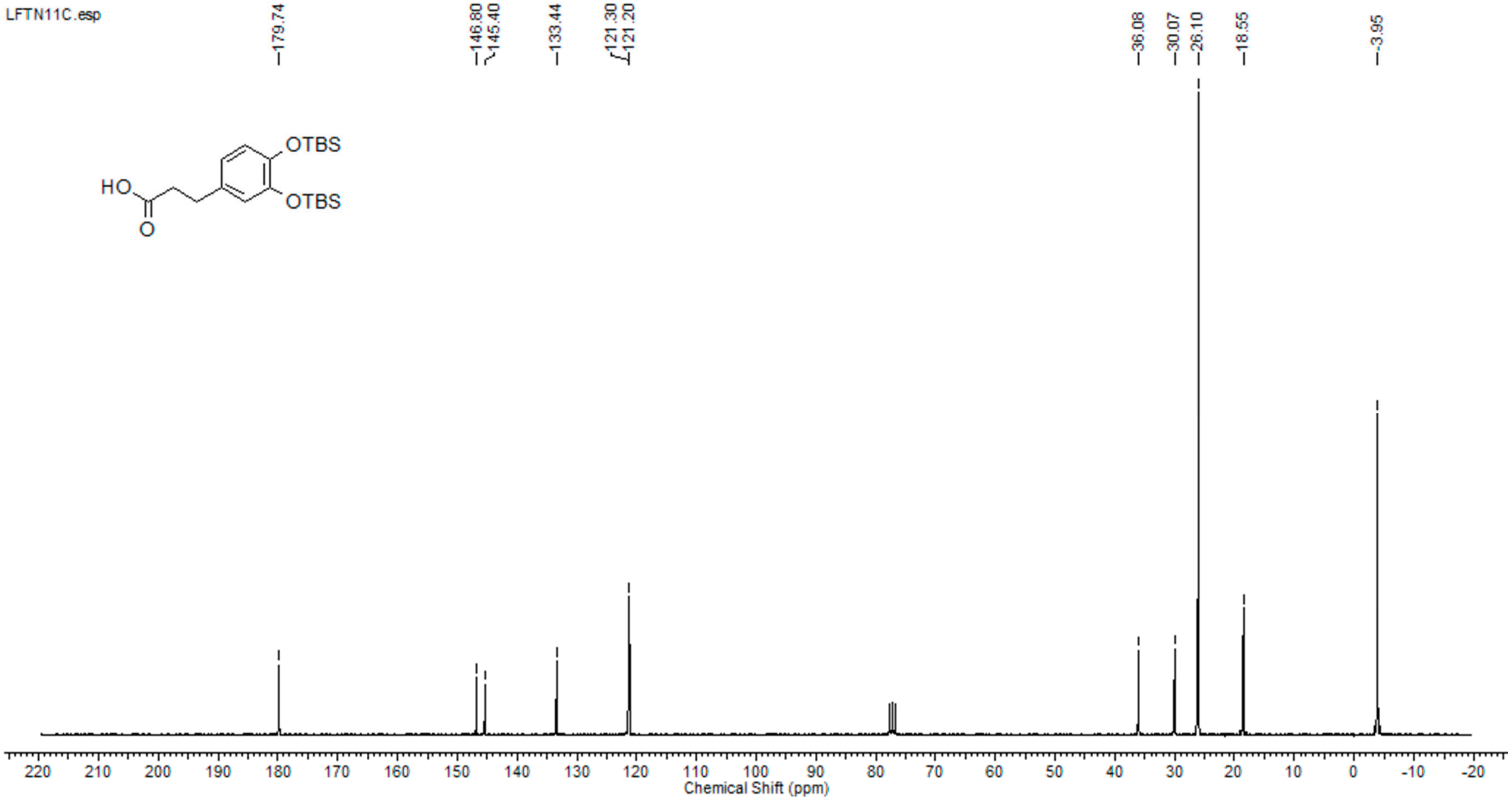

Figure S20. ^{13}C-NMR (62.9 MHz, $CDCl_3$) of compound **x**.

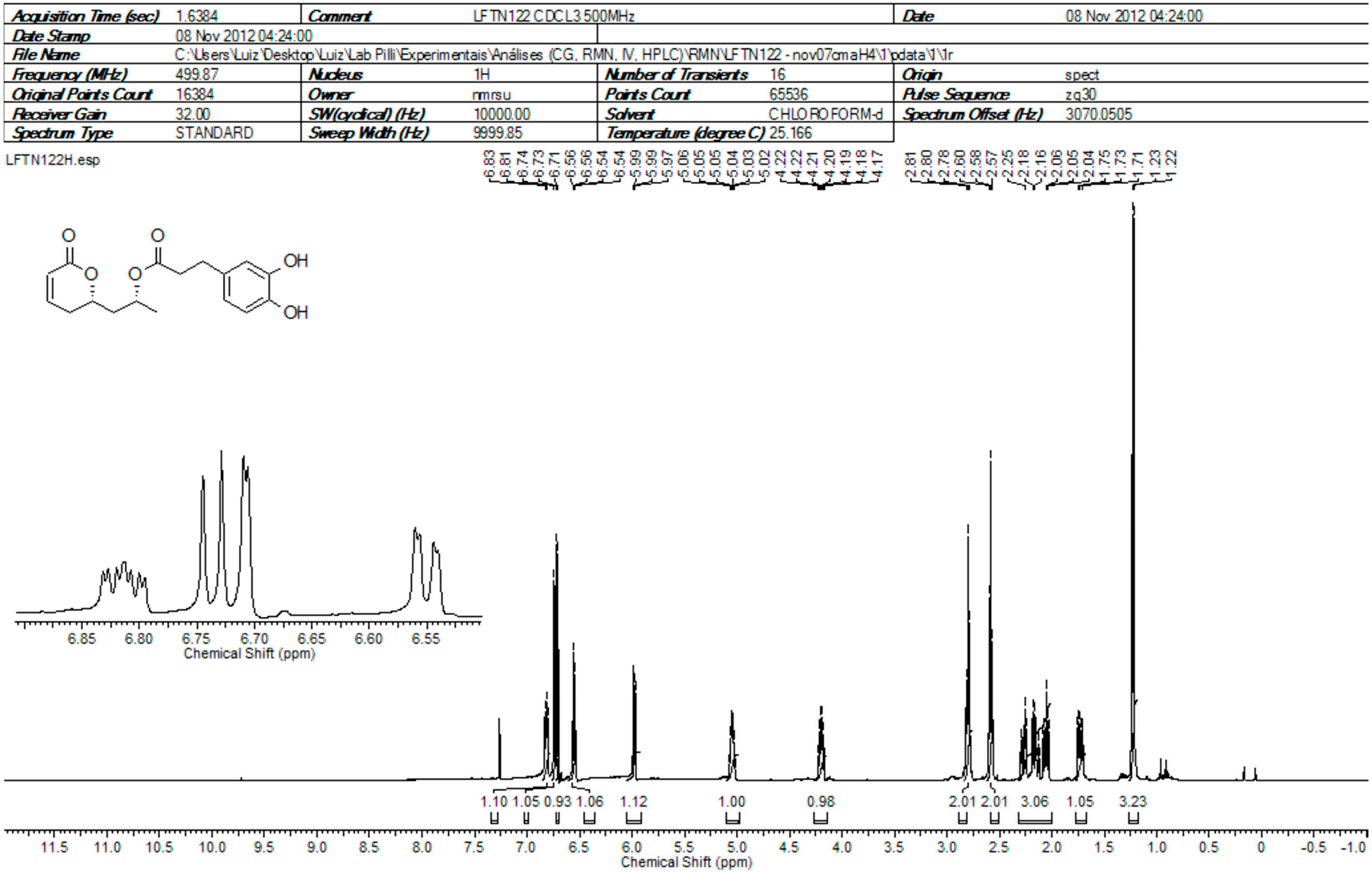

Figure S21. ^{1}H-NMR (500 MHz, $CDCl_3$) of compound **1**.

Acquisition Time (sec)	0.5505	Comment	LFTN122 CDCL3 500MHz C13			Date	08 Nov 2012 05:10:56
Date Stamp	08 Nov 2012 05:10:56						
File Name	C:\Users\Luiz\Desktop\Luiz\Lab Pilli\Experimentais\Análises (CG, RMN, IV, HPLC)\RMN\LFTN122 - nov07cmaH4\2\pdata\1\1r						
Frequency (MHz)	125.69	Nucleus	13C	Number of Transients	1024	Origin	spect
Original Points Count	16384	Owner	nmrsu	Points Count	32768	Pulse Sequence	zgpg30
Receiver Gain	2050.00	SW(cyclical) (Hz)	29761.90	Solvent	CHLOROFORM-d	Spectrum Offset (Hz)	12577.1611
Spectrum Type	STANDARD	Sweep Width (Hz)	29761.00	Temperature (degree C)	25.166		

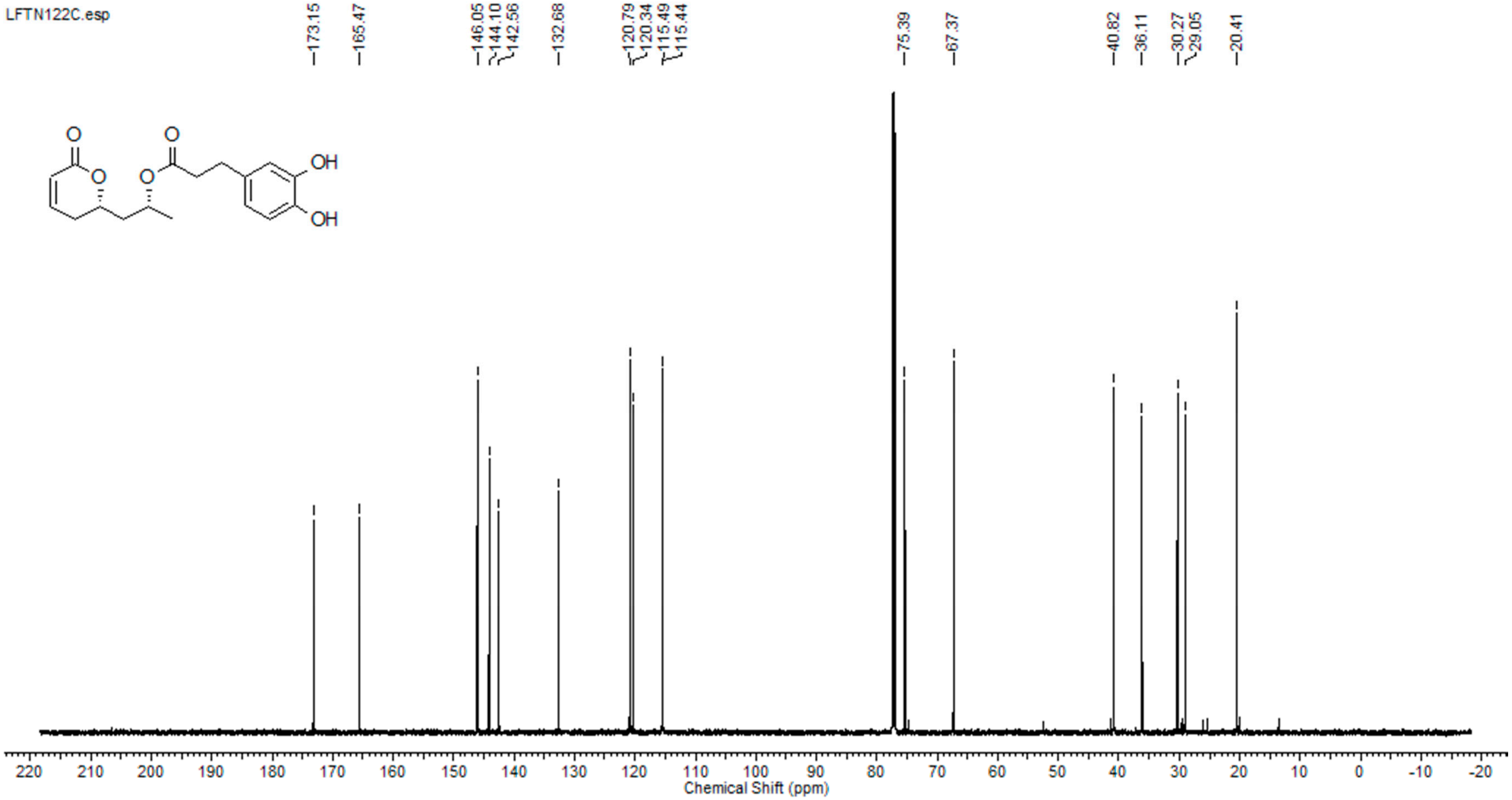

Figure S22. ^{13}C-NMR (125 MHz, $CDCl_3$) of compound **1**.

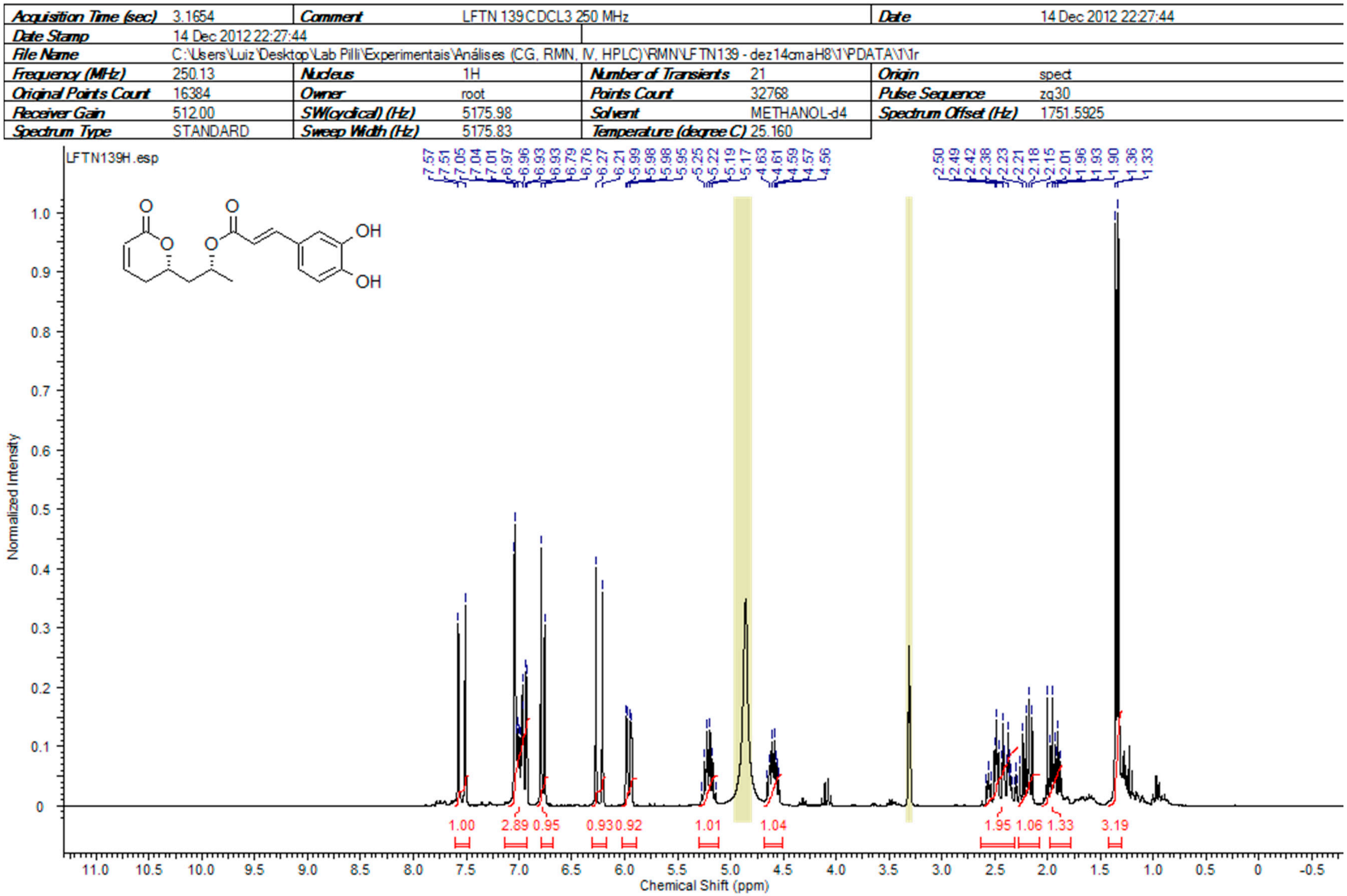

Figure S23. ^{1}H-NMR (250 MHz, methanol-d4) of compound **4**.

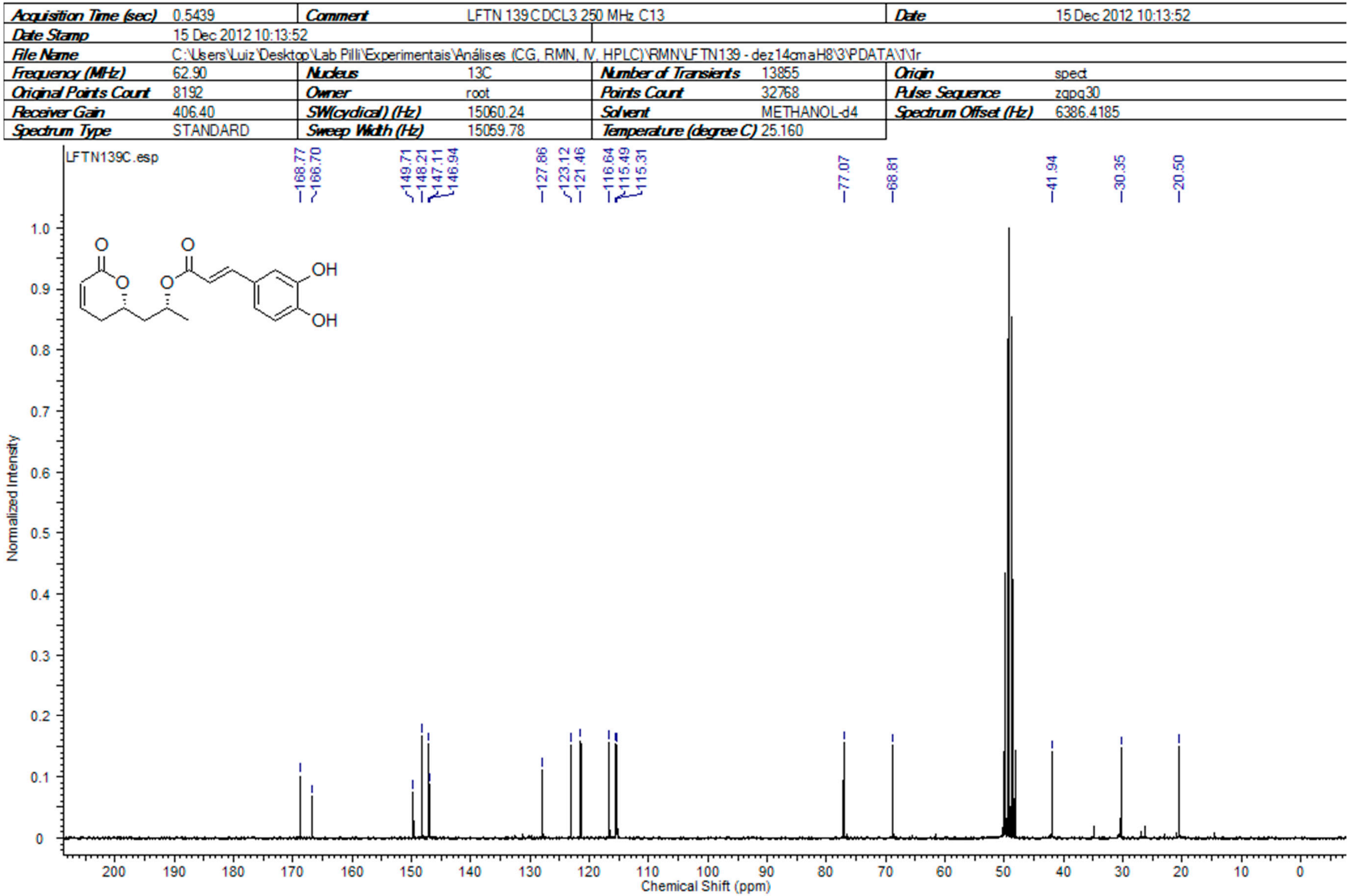

Figure S24. ^{13}C-NMR (62.9 MHz, methanol-d4) of compound **4**.
